# Anti-uPAR CAR T cells reverse and prevent aging-associated defects in intestinal regeneration and fitness

**DOI:** 10.1038/s43587-025-01022-w

**Published:** 2025-11-25

**Authors:** Onur Eskiocak, Joseph Gewolb, Vyom Shah, James A. Rouse, Saria Chowdhury, Erdogan O. Akyildiz, Inés Fernández-Maestre, Jacob A. Boyer, Aveline Filliol, Alexander S. Harris, Raditya Utama, Guangran Guo, Carolina Castro-Hernández, Emmanuella Nnuji-John, Charlie Chung, Arianna Anderson, Sara Flowers, Jill Habel, Paul B. Romesser, Ross L. Levine, Scott W. Lowe, Michel Sadelain, Semir Beyaz, Corina Amor

**Affiliations:** 1 Cold Spring Harbor Laboratory, Cold Spring Harbor, NY USA; 2 Graduate Program in Genetics, Stony Brook University, Stony Brook, NY USA; 3 Rutgers New Jersey Medical School, Newark, NJ USA; 4 Molecular and Translational Biomedicine Program, Institute of Natural and Applied Sciences, Acibadem Mehmet Ali Aydinlar University, Istanbul, Turkey; 5 Department of Molecular Biology and Genetics, Faculty of Engineering and Natural Sciences, Acibadem Mehmet Ali Aydinlar University, Istanbul, Turkey; 6 Lewis Sigler Institute for Integrative Genomics and Department of Chemistry, Princeton University, Princeton, NJ USA; 7 Ludwig Institute for Cancer Research, Princeton Branch, Princeton, NJ USA; 8 Department of Cancer Biology and Genetics. Memorial Sloan Kettering Cancer Center, New York, NY USA; 9 School of Biological Sciences, Cold Spring Harbor Laboratory, Cold Spring Harbor, NY USA; 10 Human Oncology and Pathogenesis Program, Memorial Sloan Kettering Cancer Center, New York, NY USA; 11 Department of Medicine, Weill Cornell Medical College, New York, NY USA; 12 Howard Hughes Medical Institute, Memorial Sloan Kettering Cancer Center, New York, NY USA; 13 Columbia Initiative in Cell Engineering and Therapy (CICET), Columbia University Irving Medical College, New York, NY USA; 14 Howard Hughes Medical Institute, Cold Spring Harbor Laboratory, Cold Spring Harbor, NY USA

**Keywords:** Senescence, Ageing, Cell therapies, Ageing

## Abstract

Intestinal stem cells (ISCs) drive the rapid regeneration of the gut epithelium. However, during aging, their regenerative capacity wanes, possibly through senescence and chronic inflammation, albeit little is known about how aging-associated dysfunction arises in the intestine. We previously identified the urokinase plasminogen activator receptor (uPAR) as a senescence-associated protein and developed CAR T cells able to efficiently target it. Harnessing them, here, we identify the accumulation of mostly epithelial uPAR-positive cells in the aging gut and uncover their detrimental impact on ISC function in aging. Thus, both therapeutic and prophylactic treatment with anti-uPAR CAR T cells improved barrier function, regenerative capacity, inflammation, mucosal immune function and microbiome composition in aged mice. Overall, these findings reveal the deleterious role of uPAR-positive cells on intestinal aging in vivo and provide proof of concept for the potential of targeted immune-based cell therapies to enhance tissue regeneration in aging organisms.

## Abstract (web summary)

uPAR is a senescence-associated protein, and CAR T cells targeting uPAR exert senolysis. Here Eskiocak et al. identify uPAR^+^ cells as key targets of intestinal aging and show that CAR T-mediated elimination prevents and restores age-related decline in intestinal regeneration and barrier function.

## Main

Tissue regeneration is essential for maintaining organismal homeostasis^1^. Driven by intestinal stem cells (ISCs), the intestinal epithelium exhibits one the highest rates of self-renewal^2^. However, aging considerably diminishes ISC regenerative capacity, leading to a decline in intestinal epithelial function, increased barrier permeability (‘leaky gut’) and dysbiosis^3–7^. Given the high incidence of gut disorders in older people^8^, there is a pressing need to develop strategies to rejuvenate ISC function. A number of approaches have been tested to enhance ISCs activity, including dietary modifications and small molecules, but the sustainability, efficacy in humans, safety and long-term effects of these interventions remain unclear^5,6,9–13^. Therefore, better understanding of the cellular basis for the regenerative decline of the intestinal epithelium could lead to the development of targeted and effective healthspan-promoting interventions.

Age-induced defects in intestinal fitness have been linked to a cumulative and chronic inflammatory state referred to as ‘inflammaging’^14^, which in turn further exacerbates intestinal functional decline^15^. Additionally, a key determinant of organismal aging is cellular senescence^16^. Senescence is a stress response program characterized by stable cell cycle arrest and the production of a proinflammatory senescence-associated secretory phenotype (SASP)^17^. Senescent cells accumulate with age and contribute to inflammaging and the pathophysiology of a wide range of age-related diseases^18^. How senescence and inflammaging impact tissue regeneration remains an area of very active research, with studies demonstrating substantial tissue-specific and context-specific heterogeneity^19–24^.

We recently showed that the cell surface expression of urokinase plasminogen activator receptor (uPAR) is associated with the senescence state in models of senescence acutely induced in young animals (such as oncogene induced senescence, therapy induced senescence and liver fibrosis^25^) as well as in the context of naturally aged tissues such as the liver, pancreas and adipose tissue^26^. Others have further validated and expanded these findings by showing that uPAR expression is associated with liver fibrosis, lung injury, collagen-induced arthritis and aging^27–29^. However, the presence, characteristics and functional role of uPAR^+^ cells in the context of aging tissue regeneration and intestinal biology remain unexplored.

A limitation in performing these studies has been the lack of specific and potent in vivo somatic tools that would allow to address these questions in aging as well as directly enable innovative therapeutic strategies. On this front, we recently developed the first chimeric antigen receptor (CAR) T cells able to specifically eliminate uPAR^+^ cells efficiently and safely^25,26^. CARs redirect the effector function of T cells toward a specific cell-surface antigen and are highly selective at eliminating target-expressing cells^30^. Thus, anti-uPAR CAR T cells have been shown to specifically ablate cells that express surface uPAR, and their activity in mouse models of aging and age-related diseases, such as liver fibrosis, shows decreased expression of uPAR and senescence markers in tissues and enhanced healthspan^26,27,29,31^. Interestingly, in the context of aging, anti-uPAR CAR T cells can persist and develop long-term memory, mediating also prophylactic effects^26^. However, to date, the potential impact of anti-uPAR CAR T cells on stem cell activity and tissue regeneration, whose degeneration is key hallmark of the aging process, remains unexplored.

Here, we studied the presence and functional impact of uPAR^+^ cells on intestinal regeneration and fitness during physiological aging. For this, we harnessed CAR T cells as a potent and specific tool to ablate uPAR^+^ cells in the small intestines of aging mice, and in so doing, uncovered their therapeutic and prophylactic potential for promoting tissue regeneration.

## Results

### Aging mouse and human small intestines accumulate uPAR^+^ cells

**Fig. 1:**
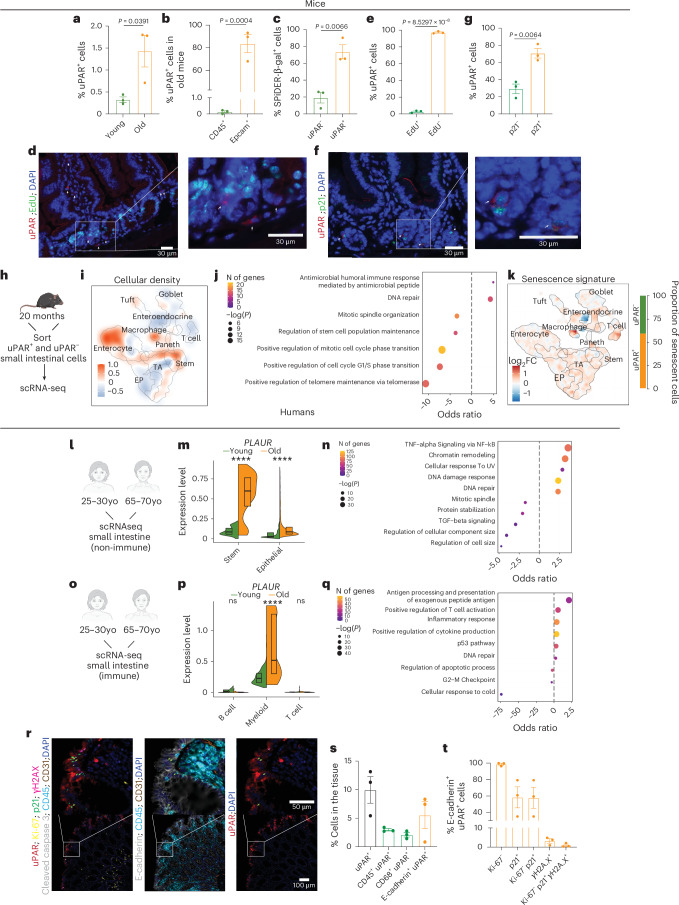
uPAR^+^ cells accumulate in aging in murine and human intestines. **a**, Surface uPAR expression as determined by flow cytometry on isolated intestinal crypts from young (3 months) and old (20 months) mice (*n* = 3 per group). **b**, Percentage of uPAR^+^ cells that are either EpCAM^+^ CD45^−^ or EpCAM^−^ CD45^+^ as determined by flow cytometry on isolated intestinal crypts from old (20 months) mice (*n* = 3 per group). **c**, Surface uPAR expression in SPiDER-β-gal^+^ cells as determined by flow cytometry on isolated intestinal crypts from old (20 months) mice (*n* = 3 per group). **d**, Representative co-immunofluorescence of uPAR (red) and EdU (green) in the proximal jejunum of aged (20 months old mice) (*n* = 3 mice). White arrows signal uPAR^+^ EdU^−^ cells. **e**, Percentage of uPAR^+^ cells that are EdU^+^ or EdU^−^ in the proximal jejunum of aged (20 months) mice (*n* = 3 mice). **f**, Representative co-immunofluorescence of uPAR (red) and p21 (green) in the proximal jejunum of aged (20 months) mice (*n* = 3 mice). White arrows signal uPAR^+^ cells. **g**, Percentage of uPAR^+^ cells that are p21^+^ or p21^−^ in the proximal jejunum of aged (20 months) mice (*n* = 3 mice). **h**–**k**, uPAR^+^ and uPAR^−^ cells from isolated intestinal crypts of duodenum, jejunum and ileum (whole small intestine) from old (20 months) mice were FACS sorted and subjected to scRNA-seq (*n* = 4 mice per group pooled into two replicates per group). **i**, Uniform Manifold Approximation and Projection (UMAP) visualization of small intestinal cell types generated by 10X chromium protocol. Color scale indicates differences in density of cellular populations between uPAR^+^ and uPAR^−^ cells. **j**, Pathway analysis using enrichR comparing differentially expressed genes between uPAR^+^ versus uPAR^−^ cells in scRNA-seq data. Size scale represents number of genes in each ontology, and color scale represents degree of significance. **k**, UMAP visualization of small intestinal cell types generated by 10X chromium protocol. Color scale indicates log_2_ fold change (log2FC) in senescence signature^34^ between uPAR^+^ and uPAR^−^ cells. Right: quantification of the proportion of uPAR^+^ and uPAR^−^ cells contributing to the senescence signature. **l**–**n**, scRNA-seq of small intestinal non-immune cell types in the whole small intestine of young (25–30 years old) and old (65–70 years old) subjects generated by 10X chromium protocol^35^ (*n* = 1 per group). **m**, Split-violin plot indicates the expression level of *PLAUR* in the ISC and epithelial lineage. Boxplots display median (center line) and interquartile range (box). **n**, Pathway analysis using enrichR comparing differentially expressed genes between non-immune *PLAUR*^+^ vs *PLAUR*^−^ cells in scRNA-seq data. Size scale represents number of genes in each ontology, and color scale represents degree of significance. **o**–**q**, scRNA-seq of small intestinal immune cell types in the whole small intestine of young (25–30 years old) and old (65–70 years old) subjects generated by 10X chromium protocol^35^ (*n* = 1 per group). **p**, Split-violin plot indicates the expression level of *PLAUR* in B cells, myeloid cells and T cells. Boxplots display median (center line) and interquartile range (box). **q**, Pathway analysis using enrichR comparing differentially expressed genes between immune *PLAUR*^+^ versus *PLAUR*^−^ cells in scRNA-seq data. Size scale represents number of genes in each ontology, and color scale represents degree of significance. **r**, Multiplex immunofluorescence of uPAR, ki-67, p21, γH2A.X, cleaved caspase-3, CD45, CD31, E-cadherin and DAPI in human intestinal samples from subjects aged 51–91 years (*n* = 3 subjects). Green arrows highlight uPAR^+^ p21^+^ cells, pink arrows highlight uPAR^+^ γH2A.X^+^ cells. **s**, Percentage of cells in the tissues from t that are uPAR^+^ CD45^+^ uPAR^+^, CD68^+^uPAR^+^ or E-Cadherin^+^ uPAR^+^ (*n* = 3 subjects). **t**, Percentage of E-Cadherin^+^ uPAR^+^ from t that are Ki-67^−^, p21^+^, Ki-67^−^ and p21^+^, γH2A.X^+^, or Ki-67^−^, p21^+^ and γH2A.X^+^ (*n* = 3 subjects). Shown are results of one independent experiment (**a–t**). Data are mean ± standard error of the mean (s.e.m.) (**a–c**,**e**,**g**,**s–t**). Significance was determined using a two-tailed unpaired Student’s *t*-test (**a–c**,**e**,**g**), two-tailed Fischer’s exact test (**j**,**n**,**q**) or two-tailed Wilcoxon rank-sum test (*P < 0.05,**P < 0.01, ***P < 0.001, ****P < 0.0001 (**m**,**p**). Illustration was created with Biorender.com (**h**). Source data

**Extended Data Fig. 1:**
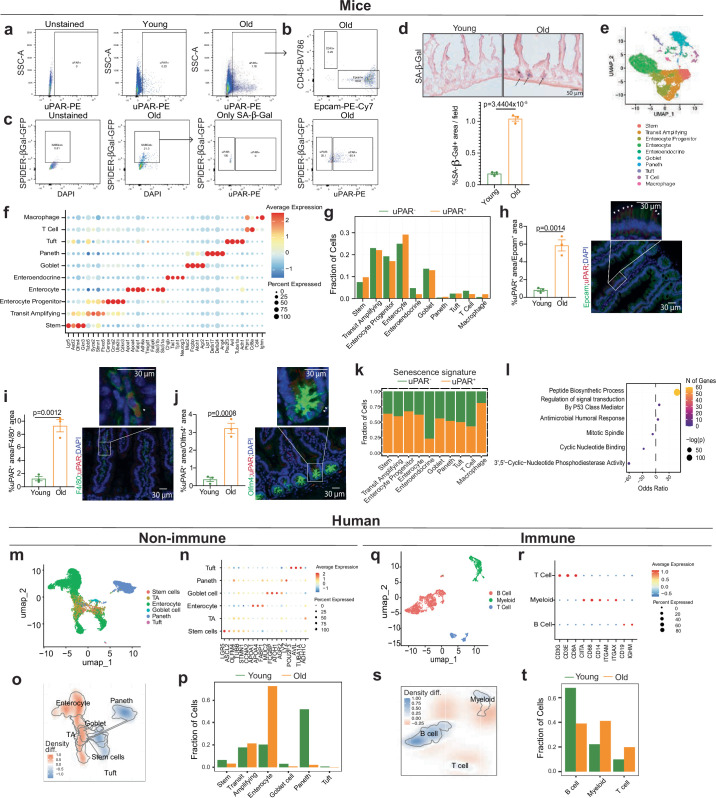
Age-dependent accumulation and characteristics of uPAR^+^ cells in murine and human small intestines. **a**, Representative gating strategy from Fig. 1a. **b**, Representative gating strategy from Fig. 1b. **c**, Representative gating strategy from Fig. 1c. **d**, Representative SA-β-gal staining of proximal jejunum of young (3 months) and old (20 months) mice and quantification (n=3 per group). **e-g,k-i**, uPAR^+^ and uPAR^−^ cells from isolated intestinal crypts of whole small intestine from 20 months old mice were FACS sorted and subjected to scRNA-seq (n=4 mice per group pooled into 2 replicates per group).**e**, UMAP visualization of small intestinal cell types generated by 10X chromium protocol. Colors indicate the 10 different intestinal epithelial lineages. **f**, Dot plot showing the 40 signature gene expressions across the 10 lineages. The size of the dots represents the proportion of cells expressing a particular marker, and the color scale indicates the mean expression levels of the markers (log1p transformed). **g**, Fraction of cells for each of the different cell types shown in e in uPAR+ and uPAR- cells. **h-i**. Percentage of uPAR^+^ area per EpCAM^+^ (**h**) or F4/80^+^ (**i**) or Olfm4^+^ (**j**) area per field as determined by immunofluorescence in the proximal jejunum of young (3 months) and old (20 months) mice (n=3 per group). Right: Representative immunofluorescence staining of EpCAM or F4/80 or Olfm4 (green), uPAR (red) and DAPI (blue) from old (20 months old) mice. White arrows highlight double positive cells. **k**, Stacked bar plot depicting fraction of cells expressing the senescence signature in Fig. 1k between uPAR+ and uPAR- cells across cell types shown in e. **l**, Pathway analysis using enrichR comparing differentially expressed genes between senescent uPAR^+^ cells versus senescent uPAR^−^ cells in scRNA-seq data. Size scale represents number of genes in each ontology and color scale represents degree of significance. **m-p** scRNA-seq of small intestinal non-immune cell types in the whole small intestine of young (25–30 years old) and old (65–70 years old) subjects generated by 10X chromium protocol^35^ (n=1 per group). **m**, UMAP visualization of human cell types in small intestine generated by 10X chromium protocol. Colors indicate the 6 different intestinal epithelial lineages. **n**, Dot plot showing the 18 signature gene expressions across the 6 lineages. The size of the dots represents the proportion of cells expressing a particular marker, and the color scale indicates the mean expression levels of the markers (log1p transformed). **o**, UMAP visualization of human non-immune cell types in small intestine generated by 10X chromium protocol. Color scale indicates differences in density of cellular populations between old (65–70 years old) and young (25–30 years old) subjects. **p**, Fraction of cells in each of the 6 different populations from young (25–30 years old) and old (65–70 years old) subjects. **q-t** scRNAseq of small intestinal immune cell types in the whole small intestine of young (25–30 years old) and old (65–70 years old) subjects generated by 10X chromium protocol^35^ (n=1 per group). **q**, UMAP visualization of human cell types in small intestine generated by 10X chromium protocol. Colors indicate the 3 different intestinal immune lineages. **r**, Dot plot showing the 10 signature gene expressions across the 3 lineages. The size of the dots represents the proportion of cells expressing a particular marker, and the color scale indicates the mean expression levels of the markers (log1p transformed). **s**, UMAP visualization of human immune cell types in small intestine generated by 10X chromium protocol. Color scale indicates differences in density of cellular populations between old (65–70 years old) and young (25–30 years old) subjects. **t**, Fraction of cells in each of the 3 different populations from young (25–30 years old) and old (65–70 years old) subjects. Results of 1 independent experiment (**a–t**). Data are mean ± s.e.m. (**d,h-j**). Two-tailed unpaired Student’s t-test (**d,h-j**). Two-tailed Fischer’s exact test (**l**). Source data

To investigate whether uPAR^+^ cells accumulate during physiological aging in the small intestine, we performed flow cytometry to detect cells that express surface uPAR protein in the proximal jejunum of young (3-month-old) and old (20-month-old) mice. We found a significant increase in the percentage of uPAR^+^ cells with aging (Fig. 1a and Extended Data Fig. 1a), which were mostly of epithelial origin (Fig. 1b and Extended Data Fig. 1b). Given our previous observation that uPAR expression in other tissues and cell types is associated with senescence^25,26^, we performed senescence-associated beta-galactosidase (SA-β-gal) staining in the proximal jejunum of young and old mice and found a significant age-dependent increase in the number of SA-β-gal^+^ cells, with SA-β-gal^+^ cells being significantly enriched in surface uPAR expression (~73% double positive) (Fig. 1c and Extended Data Fig. 1c,d). Beyond SA-β-gal, uPAR^+^ cells in the small intestine presented additional features traditionally associated with the senescence program^32^, such as absence of proliferation in ~97% of cells (as determined by EdU pulse labeling) (Fig. 1d,e) and co-staining with p21 in ~70% of cells (Fig. 1f,g).

To better characterize the cell types that upregulate uPAR surface expression in this setting, we isolated cells expressing surface uPAR (uPAR^+^) and those that do not (uPAR^−^) cells from aged (20-month-old) entire small intestine (duodenum, jejunum and ileum) through fluorescence-activated cell sorting (FACS) and then performed single-cell RNA sequencing (scRNA-seq) (Fig. 1h). We profiled 9430 uPAR^+^ and 7379 uPAR^−^ individual cells. Using unsupervised clustering and marker-based cell labelling^33^, we assigned 10 different cell types which were visualized with Uniform Manifold Approximation and Projection (UMAP) (Extended Data Fig. 1e,f). Analysis of the different populations for uPAR expression indicated that stem cells, enterocytes and macrophages were the most prominent uPAR-expressing populations in the aged small intestine (Fig. 1i and Extended Data Fig. 1g). Histological analysis confirmed the presence of uPAR^+^ stem cells, enterocytes and macrophages in both young and old animals; with the latter having significantly higher proportions of these populations (Extended Data Fig. 1h–j). To understand their characteristics, we compared the gene expression profile of uPAR^+^ and uPAR^−^ cells and found that uPAR^+^ cells were significantly enriched in terms related to DNA repair and immune response, whereas cell proliferation was significantly downregulated compared to uPAR^−^ cells (Fig. 1j). Because these features are reminiscent of the senescence program, we performed computational analysis of the expression of the SenMayo signature of senescence^34^ and found that around 60% of the cells identified as ‘senescent’ by SenMayo belonged to those expressing surface protein uPAR expression (Fig. 1k and Extended Data Fig. 1k). Among the computationally identified senescent cells, those that were uPAR^+^ appeared particularly enriched in terms related to p53 activity, immune response and decreased proliferation compared to those that were uPAR^−^ (Extended Data Fig. 1l).

To explore whether a similar accumulation of uPAR^+^ cells takes place in human intestines, we surveyed scRNA-seq data from samples of non-immune cell types from the duodenum, jejunum and ileum in old (65–70 years) and young (25–30 years) individuals^35^ (Fig. 1l–n and Extended Data Fig. 1m–p) and immune cell types from the duodenum, jejunum and ileum in old (65–70 years) and young (25–30 years) individuals^35^ (Fig. 1o–q and Extended Data Fig. 1q–t). Although we were limited to the analysis of cells that express *PLAUR* (the gene encoding uPAR), rather than of cells that express surface protein uPAR protein, we observed a significant upregulation of *PLAUR* mRNA in aged stem, epithelial and myeloid populations (Fig. 1m,p). Similar to murine small intestine uPAR^+^ cells, the transcriptional profile of these human *PLAUR*^*+*^ cells was enriched in terms related to DNA damage repair and inflammation and downregulated in terms related to proliferation (Fig. 1n,q). To validate these results at the protein level, we performed multiplex immunofluorescence staining in aged human intestines. Although we were limited to the analysis of colon because of biospecimen availability, we found that uPAR^+^ cells in aged human intestines were also preferentially epithelial rather than immune (Fig. 1r,s) and also exhibited absence of proliferation and expression of p21 (Fig. 1r,t). Although there were few spontaneous γH2A.X^+^ cells present in the aged intestines, some uPAR^+^ cells were also γH2A.X^+^ (Fig. 1r,t).

Taken together, these results indicate that uPAR^+^ cells accumulate in the intestines of both mice and humans during physiological aging. Intestinal uPAR^+^ cells are preferentially epithelial and are characterized by an enrichment in the expression of terms related to cell cycle arrest, DNA repair and inflammation.

### In vivo targeting of uPAR^+^ cells improves age-associated defects in intestinal epithelial barrier integrity

To functionally interrogate the physiological consequences of this age-dependent accumulation of uPAR^+^ cells in the intestine in vivo, we harnessed CAR T cells to eliminate them. For this, we used second-generation murine uPAR targeting CAR T cells (m.uPAR-m.28z) that express a single-chain variable fragment recognizing mouse uPAR and have mouse CD28 as a costimulatory domain^25,31^. uPAR CAR T cells are safe and selectively eliminate uPAR^+^ cells in vivo, including in the context of aging, where a single infusion has been shown to lead to long-term persistence of the senolytic CAR T cells and their effects^25,26^.

**Fig. 2:**
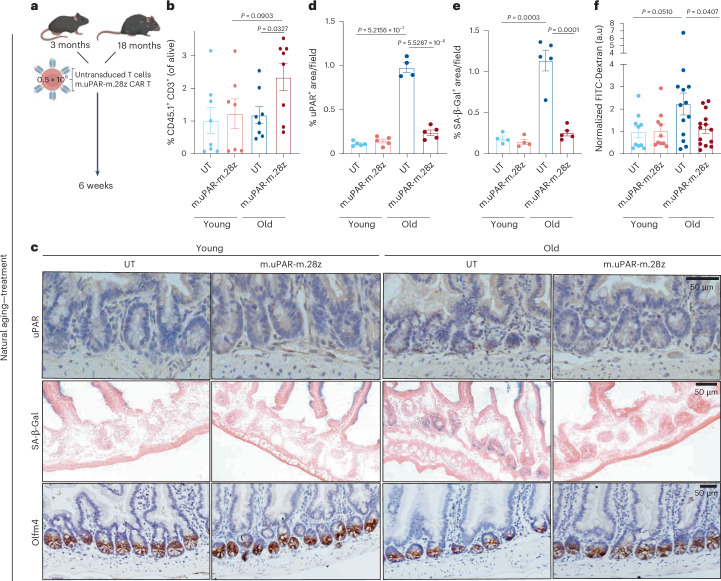
Therapeutic treatment with uPAR targeting CAR T cells rescue age-related defects in intestinal epithelium integrity. **a**, Experimental scheme for a–f: Young (3 months) and old (18 months) mice were treated with 0.5 × 10^6^ untransduced T cells (UT) or uPAR CAR T cells (m.uPAR-m.28z). Mice were harvested 6 weeks after infusion. **b**, Percentage of CD45.1 and CD3 double positive cells in the intestinal crypts as assayed by flow cytometry (*n* = 8 for young UT, *n* = 7 young m.uPAR-m.28z, *n* = 8 for old UT, *n* = 8 old m.uPAR-m.28z). **c**, Representative staining of uPAR, SA-β-gal and Olfm4 in proximal jejunum. **d**, Percentage of histological area with uPAR^+^ cells per field as determined by immunohistochemistry in the proximal jejunum (*n* = 5 for UT and m.uPAR-m.28z young; *n* = 5 m.uPAR-m.28z old; *n* = 4 for UT old). **e**, Percentage of histological area with SA- β-gal^+^ cells in the proximal jejunum (*n* = 4 for UT and m.uPAR-m.28z young; *n* = 5 UT old; *n* = 5 for m.uPAR-m.28z old). **f**, Normalized plasma levels of FITC-Dextran 4 h after oral gavage (*n* = 10 for UT and m.uPAR-m.28z young; *n* = 13 for UT old; *n* = 14 for m.uPAR-m.28z old). Shown are results of two independent experiments (**b**,**f**) or one independent experiment (**c–e**). Data are mean ± s.e.m. (**b**,**d–f**). Significance was determined using a two-tailed unpaired Student’s *t*-test (**b**,**d–f**). Illustration was created with Biorender.com (**a**). Source data

**Extended Data Fig. 2:**
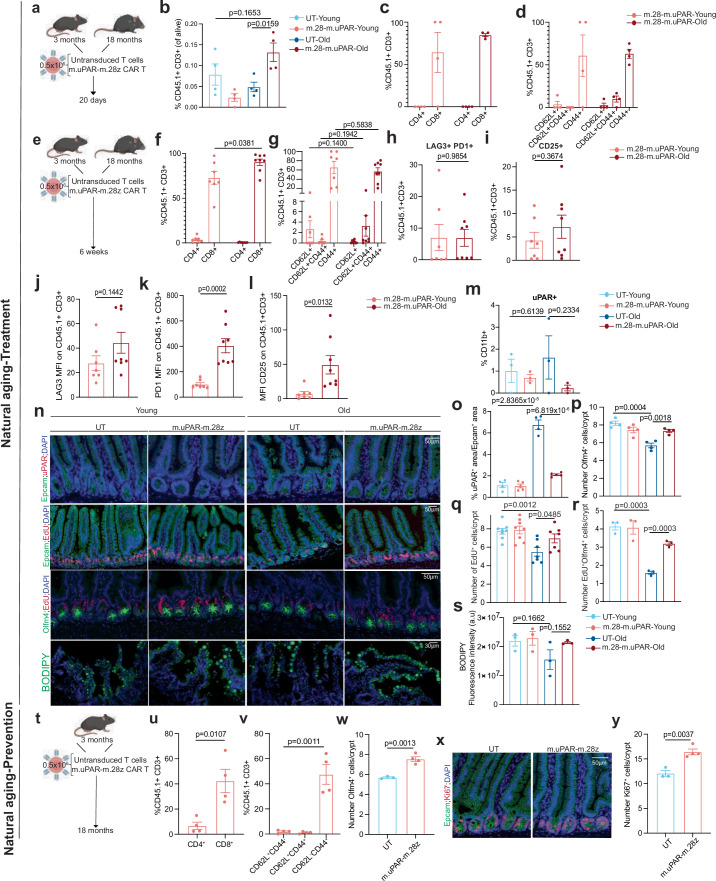
Profile and effect of uPAR CAR T cells in aged small intestine. **a**, Experimental scheme for b–d: Young (3 months) and old (18 months) mice were treated with 0.5x10^6 untransduced T cells (UT) or uPAR CAR T cells (m.uPAR-m.28z). Mice were harvested 20 days after infusion. **b**, Percentage of CD45.1^+^ CD3^+^ cells in the intestinal crypts. (n=4 per group) as assayed by flow cytometry. **c**, Percentage of CD4^+^ or CD8^+^ cells from CD45.1^+^ CD3^+^ cells in the intestinal crypts. (n=4 per group). **d**, Percentage of CD62L, CD44, and CD62L and CD44 positive cells from CD45.1^+^ CD3^+^ cells in the intestinal crypts. (n=4 per group). **e**, Experimental scheme for f-s: Young (3 months) and old (18 months) mice were treated with 0.5x10^6 untransduced T cells (UT) or uPAR CAR T cells (m.uPAR-m.28z). Mice were harvested 6 weeks after infusion. **f**, Percentage of CD4^+^ or CD8^+^ cells from CD45.1^+^ CD3^+^ cells in the intestinal crypts (n=7 for young m.uPAR-m.28z and n=8 for old m.uPAR-m.28z). **g**, Percentage of CD62L, CD44, and CD62L and CD44 positive cells from CD45.1^+^ CD3^+^ cells in the intestinal crypts (n=7 for young m.uPAR-m.28z and n=8 for old m.uPAR-m.28z). **h**, Percentage of LAG3 and PD1 double positive cells from CD45.1^+^ CD3^+^ cells in the intestinal crypts (n=7 for young m.uPAR-m.28z and n=8 for old m.uPAR-m.28z). **i**, Percentage of CD25 positive cells from CD45.1^+^ CD3^+^ cells in the intestinal crypts. (n=7 for young m.uPAR-m.28z and n=8 for old m.uPAR-m.28z). **j**, Median Fluorescence Intensity (MFI) of LAG3 on CD45.1^+^ CD3^+^ cells in the intestinal crypts (n=7 for young m.uPAR-m.28z and n=8 for old m.uPAR-m.28z). **k**, Median Fluorescence Intensity (MFI) of PD1 on CD45.1^+^ CD3^+^ cells in the intestinal crypts (n=7 for young m.uPAR-m.28z and n=8 for old m.uPAR-m.28z). **l**, Median Fluorescence Intensity (MFI) of CD25 on CD45.1^+^ CD3^+^ cells in the intestinal crypts (n=7 for young m.uPAR-m.28z and n=8 for old m.uPAR-m.28z). **m**, Percentage of uPAR^+^ cells from CD11b^+^ cells in the intestinal crypts. (n=3 per group).**n**, Representative immunofluorescence staining of EpCAM (green), uPAR (red) and DAPI (blue); EpCAM (green), EdU (red) and DAPI (blue); Olfm4 (green), EdU (red), DAPI (blue) and BODIPY (green) uptake of proximal jejunum. **o**, Percentage of uPAR^+^ EpCAM^+^ area of total EpCAM+ area (n=4 for young UT, n=5 for young m.uPAR-m.28z, n=4 for old UT, n=5 for old m.uPAR-m.28z). **p**, Quantification of number of Olfm4^+^ cells per intestinal crypt in the proximal jejunum in samples from Fig. 2c.(n=4 per group). **q**, Quantification of number of EdU^+^ cells per intestinal crypt in the proximal jejunum (n=8 for UT and m.uPAR-m.28z young and n=7 for UT and m.uPAR-m.28z old). **r**, Quantification of number of EdU^+^ Olfm4^+^ cells per intestinal crypt in the proximal jejunum (n=3 per group). **s**, Quantification of the fluorescence intensity of BODIPY in jejunum (n=3 per group). **t**, Experimental scheme for u-y: Young (3 months) mice were treated with 0.5x10^6 untransduced T cells (UT) or uPAR CAR T cells (m.uPAR-m.28z). Mice were harvested 15 months after infusion when the mice were 18 months old. **u**, Percentage of CD4^+^ or CD8^+^ cells from CD45.1^+^ CD3^+^ cells in the intestinal crypts of m.uPAR-m.28z mice (n=4). **v**, Percentage of CD62L, CD44, and CD62L and CD44 positive cells from CD45.1^+^ CD3^+^ cells in the intestinal crypts of m.uPAR-m.28z mice (n=4). **w**, Quantification of number of Olfm4^+^ cells per intestinal crypt in the proximal jejunum (n=3 for UT and n=4 for m.uPAR-m.28z). **x**, Representative immunofluorescence staining of EpCAM (green), EdU (red) and DAPI (blue) in the proximal jejunum. **y**, Quantification of number of Ki-67^+^ cells per intestinal crypt in the proximal jejunum (n=3 for UT and n=4 for m.uPAR-m.28z). Results from 2 independent experiments (**f-l,q**). Results from 1 independent experiment (**b–d,m-p,r-y**). Data are mean ± s.e.m. (**b–d,f-m,o-s,u-w,y**). Two-tailed unpaired Student’s t-test (**b–d,f-m,o-s,u-w,y**). Illustration created with Biorender.com (**a,e,t**). Source data

Thus, we performed studies in syngeneic mouse strains in which uPAR CAR T cells or control untransduced T cells (herein designated UT) from CD45.1 mice were intravenously infused into CD57BL/6 CD45.2 young (3 months old) and old (18–20 months old) mice (Fig. 2a). We used a dose of 0.5 × 10^6^ CAR^+^ cells, which we have observed to be optimal for senolytic efficacy and safety^25,26^. Importantly, in this setting and at this dose, uPAR CAR T cells were initially detected by flow cytometry in the intestinal epithelium of the mice 20 days after infusion, where they were present in higher numbers in aged animals and predominantly presented a cytotoxic effector/effector-memory T cell phenotype (CD8^+^ CD44^+^ CD62L^−^) (Extended Data Fig. 2a–d). uPAR CAR T cells expanded over time in aged animals and were detected at higher percentages in the intestinal epithelium 6 weeks after infusion, where they still presented an active cytotoxic effector T cell phenotype with low levels of exhaustion markers (Fig. 2b and Extended Data Fig. 2e–l), suggesting that they were recognizing uPAR^+^ cells in this tissue. Indeed, administration of uPAR CAR T cells led to a decrease in the number of uPAR^+^ cells (both epithelial and myeloid) as well as a reduction in the number of SA-β-Gal^+^ cells in the small intestines of aged uPAR CAR T-treated mice versus those that received control UT cells (Fig. 2c–e and Extended Data Fig. 2m–o).

Phenotypically, the elimination of uPAR^+^ cells led to improvements in age-associated defects in intestinal epithelial barrier integrity. Thus, treatment with uPAR CAR T cells in aged mice significantly rescued age-induced increased intestinal permeability, or ‘leaky gut’^36^, as measured by significantly decreased plasma levels of FITC-Dextran 4 h after oral administration in aged uPAR CAR T treated mice as compared with aged UT-treated animals (Fig. 2f). Histological analyses revealed that administration of uPAR CAR T cells to aged mice significantly increased the number of stem cells and proliferating (EdU^+^) cells in the intestinal crypts (Fig. 2c and Extended Data Fig. 2n,p–r). In addition, we observed modest improvements in the lipid absorption capacity of aged enterocytes (Extended Data Fig. 2s).

**Extended Data Fig. 3:**
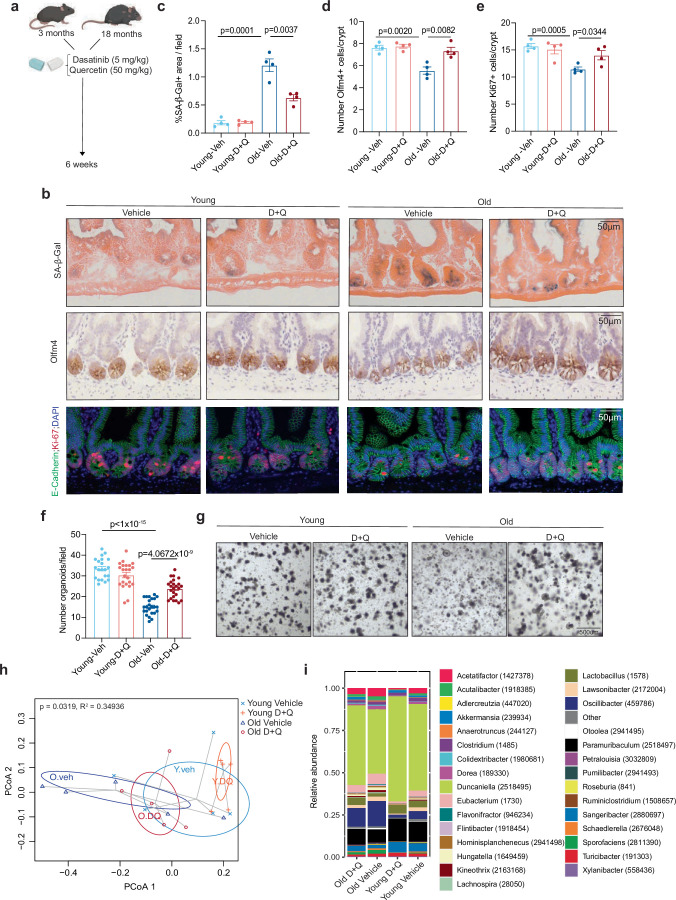
Treatment with dasatinib and quercetin improves features of intestinal aging. **a**, Experimental scheme for b-i. Young (3 months) and old (18 months) mice were treated with dasatinib (5mg/kg) and quercetin (50mg/kg) or vehicle two times a week for 6 weeks. **b**, Representative SA-β-Gal staining, immunohistochemistry of Olfm4 and immunofluorescence of E-cadherin (green), KI-67 (red) and DAPI (blue) of proximal jejunum. **c**, Percentage of histological area with SA- β-gal^+^ cells in the proximal jejunum (n=4 per group). **d**, Number of Olfm4^+^ cells per crypt in the proximal jejunum (n=4 per group). **e**, Number of Ki-67^+^ cells per crypt in the proximal jejunum (n=4 per group). **f**, Organoid initiating capacity of intestinal crypts from young or old, vehicle or D+Q treated mice (n=4 mice per group; 4–6 replicates per mouse). **g**, Representative pictures of organoids in f. **h**, Principal coordinate analysis (PCoA) of the microbial composition in fecal samples of young and old, vehicle or D+Q treated mice after 6 weeks of treatment (n=5 per group). **i**, Relative abundance of microbial genus as determined by metagenomics analysis in each treatment condition (n=5 per group). Results of 1 independent experiment (b-i). Data are mean ± s.e.m. (**c-e,f**). Two-tailed unpaired Student’s t-test (**c-e,f**). Two-tailed PERMANOVA (**h**). Illustration created with Biorender.com (**a**). Source data

To explore whether the results observed with anti-uPAR CAR T cells could be recapitulated with other strategies aimed at eliminating cells with features of senescence, we treated young (3-month-old) and old (18-month-old) mice with the combination of dasatinib (5 mg kg^−1^) and quercetin (50 mg kg^−1^) (D + Q) twice a week for 6 weeks as reported previously^21^ (Extended Data Fig. 3a). D + Q treatment resulted in a reduction in the number of SA-β-gal^+^ cells in the aged small intestines (Extended Data Fig. 3b,c) and, similar to the effects of uPAR CAR T cells, in an increase in the number of stem cells and the number of proliferating (Ki-67^+^) cells in the intestinal crypts of aged mice (Extended Data Fig. 3b,d,e). These results provide orthogonal validation to our uPAR CAR T strategy and, taken together, both approaches suggest that cells displaying some features of the senescent program contribute to the decline in intestinal epithelial integrity with aging.

**Extended Data Fig. 4:**
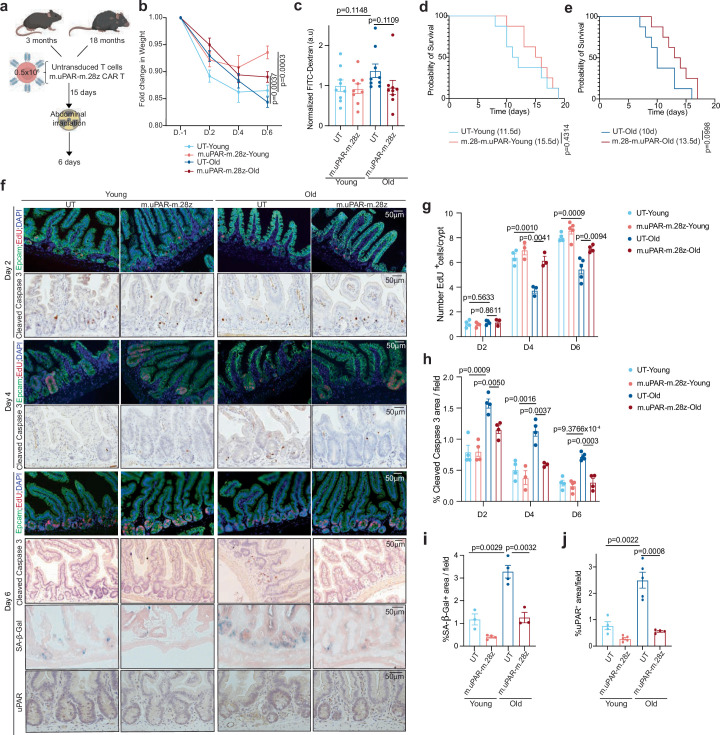
uPAR CAR T cells ameliorate the effects of abdominal irradiation in aging. **a**, Experimental scheme for b-j. Young (3 months) and old (18 months) mice were infused with 0.5x10^6 untransduced T cells (UT) or uPAR CAR T cells (m.uPAR-m.28z). 15 days after cell injection mice were subjected to abdominal irradiation with 15Gy. Mice were harvested at 2, 4 and 6 days after irradiation or their survival was monitored long-term. **b**, Fold change in weight before and after abdominal irradiation with 15Gy. (D= day). (n=10 per group). **c**, Normalized plasma levels of FITC-Dextran 4 h after oral gavage at day 6. (n=9 per group). **d**, Kaplan-Meier survival curve of young mice after abdominal irradiation. (n= 8 per group).**e**, Kaplan-Meier survival curve of old mice after abdominal irradiation. (n=8 per group). **f**, Representative immunofluorescence staining of EpCAM (green), EdU (red) and DAPI (blue), and immunohistochemistry of cleaved caspase-3 of proximal jejunum at day 2, day 4 and day 6 post irradiation and SA-β-Gal staining and immunohistochemistry of uPAR in the proximal jejunum at day 6. **g**, Quantification of number of EdU positive cells per intestinal crypt at day 2, day 4 and day 6 post irradiation. (Day 2: n=4 for UT young, n=4 m.uPAR-m.28z young, n=4 UT old, n=3 m.uPAR-m.28z old). (Day 4: n=4 for UT young, n=3 m.uPAR-m.28z young, n=3 UT old, n=3 m.uPAR-m.28z old). (Day 6: n=4 for UT young, n=5 m.uPAR-m.28z young, n=5 UT old, n=4 m.uPAR-m.28z old).**h**, Quantification of number of cleaved caspase-3 positive cells per intestinal crypt at day 2, day 4 and day 6 post irradiation. (Day 2: n=4 for UT young, n=4 m.uPAR-m.28z young, n=4 UT old, n=4 m.uPAR-m.28z old). (Day 4: n=4 for UT young, n=3 m.uPAR-m.28z young, n=4 UT old, n=3 m.uPAR-m.28z old). (Day 6: n=4 for UT young, n=5 m.uPAR-m.28z young, n=5 UT old, n=4 m.uPAR-m.28z old). **i**, Percentage of histological area with SA- β-gal^+^ cells in the proximal jejunum at day 6 post irradiation (n=3 for UT young; n=4 for m.uPAR-m.28z young; n=4 for UT old; n=3 m.uPAR-m.28z old;). **j**, Percentage of histological area with uPAR^+^ cells in the proximal jejunum at day 6 post irradiation (n=4 for UT young; n=5 for m.uPAR-m.28z young; n=5 for UT old; n=4 m.uPAR-m.28z old). Results of 2 independent experiments (**b-c**). Results of 1 independent experiment (**d-j**). Data are mean ± s.e.m. (**b-c,g-j**). Two-tailed unpaired Student’s t-test (**b-c,g-j**). Log-rank (Mantel-Cox) test (**d-e**). Illustration created with Biorender.com (**a**). Source data

To further explore the impact of uPAR CAR T cells on intestinal regeneration after injury, we challenged the mice with 15 Gy abdominal irradiation, which has been shown to elicit cytotoxicity, crypt loss and senescence in the intestinal epithelium^6^ (Extended Data Fig. 4a). Irradiation of young and old UT and uPAR CAR T treated animals induced damage to the epithelium that was followed by a regenerative phase after injury (Extended Data Fig. 4a–j). As described previously^13,37^, aged UT treated mice presented a trend toward less tolerance of abdominal irradiation than their younger counterparts exhibiting sightly increased weight loss at day 6, greater increase in intestinal permeability and decreased survival (Extended Data Fig. 4b–e). Aged UT-treated mice also presented higher levels of initial damage from irradiation than uPAR CAR T treated aged mice as measured by apoptotic cleaved caspase-3 at day 2 after irradiation, possibly indicating the aged UT-treated epithelium is more sensitive to injury than that of aged uPAR CAR T treated animals. Moreover, aged UT-treated mice exhibited slower regeneration, having lower numbers of proliferating (EdU^+^) cells at day 4 and day 6 after irradiation and higher levels of damaged apoptotic and senescent cells at day 6, whereas uPAR CAR T-treated mice exhibited faster initiation of intestinal and had higher numbers of proliferating (EdU^+^) intestinal cells at day 4 and day 6 (Extended Data Fig. 4f–j).

**Fig. 3:**
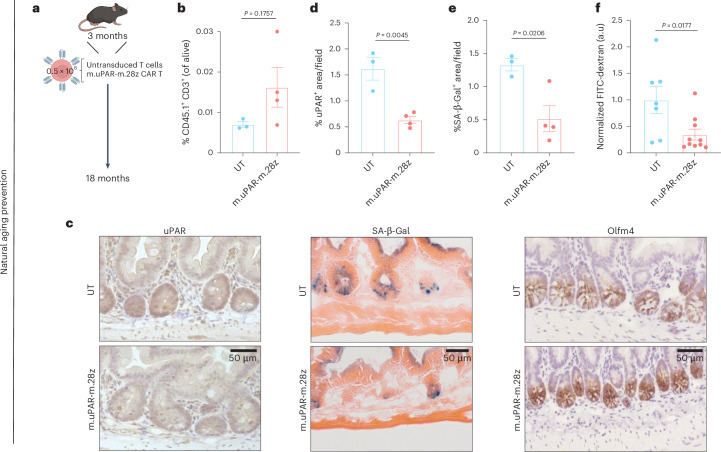
Prophylactic treatment with uPAR targeting CAR T cells rescue age-related defects in intestinal epithelium integrity. **a**, Experimental scheme for b–f: Young (3 months) mice were treated with 0.5 × 10^6^ untransduced T cells (UT) or uPAR CAR T cells (m.uPAR-m.28z). Mice were harvested 15 months after infusion at the age of 18 months. **b**, Percentage of CD45.1 and CD3 double positive cells in the intestinal crypts (*n* = 3 for UT, *n* = 4 for m.uPAR-m.28z). **c**, Representative staining of uPAR, SA-β-gal and Olfm4 in proximal jejunum. **d**, Percentage of histological area with uPAR^+^ cells per field as determined by immunohistochemistry in the proximal jejunum (*n* = 3 for UT, *n* = 4 for m.uPAR-m.28z). **e**, Percentage of histological area with SA- β-gal^+^ cells in the proximal jejunum (*n* = 3 for UT, *n* = 4 for m.uPAR-m.28z). **f**, Normalized plasma levels of FITC-Dextran 4 h after oral gavage (*n* = 7 for UT, *n* = 10 for m.uPAR-m.28z). Shown are results of two independent experiments (**f**) or one independent experiment (**b–e**). Data are mean ± s.e.m. (**b**,**d–f**). Significance was determined by two-tailed unpaired Student’s *t*-test (**b,d–f**). Illustration was created with Biorender.com (**a**). Source data

Unlike other approaches, CAR T cells have the potential to develop long-term persistence^38^. In previous work we have shown that administration of uPAR CAR T cells to young animals resulted in their presence for over a year^26^. To study how prophylactic treatment with uPAR CAR T cells would impact intestinal function upon aging, we infused a single dose of 0.5 × 10^6^ uPAR CAR^+^ cells (or UT controls) generated from CD45.1^+^ T cells into young (3-month-old) CD45.2^+^ mice (Fig. 3a). Fifteen months later, when the mice reached 18 months old, the infused uPAR CAR T cells remained detectable in the intestinal epithelium, where they presented an effector/effector-memory phenotype (CD8^+^, CD44^+^, CD62L^−^), suggesting ongoing recognition of uPAR^+^ cells in this tissue (Fig. 3b and Extended Data Fig. 2t–v). Concordantly, prophylactically uPAR CAR T treated mice presented significantly lower percentages of uPAR^+^ and SA-β-gal^+^ cells in the small intestines upon aging (Fig. 3c–e). Phenotypically, this resulted in significantly decreased intestinal permeability (Fig. 3f) as well as an increase in the number of stem cells and proliferating (EdU^+^) cells in the intestinal crypts of the mice upon aging (Fig. 3c and Extended Data Fig. 2w–y).

Overall, these data suggest that ablating uPAR^+^ cells or preventing their accumulation during aging significantly ameliorates age-associated deterioration of intestinal epithelial barrier integrity, ISC number and proliferative capacity.

### Prophylactic or therapeutic treatment with uPAR CAR T cells improves regenerative capacity of aged ISCs

**Fig. 4:**
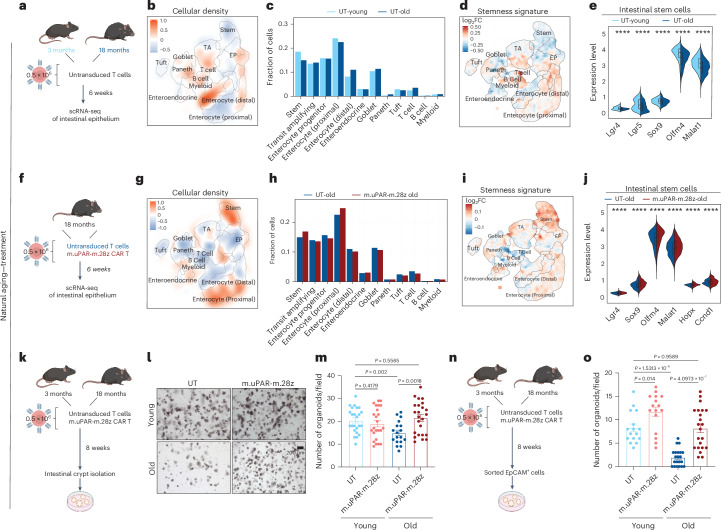
Therapeutic uPAR CAR T cells rejuvenate ISCs. **a–j**, Young (3 months) and old (18 months) mice were treated with 0.5 × 10^6^ untransduced T cells (UT) or uPAR CAR T cells (m.uPAR-m.28z). Mice were harvested 6 weeks after infusion, and scRNA-seq was performed from whole small intestine: duodenum, jejunum and ileum (*n* = 4 mice per group pooled into two replicates per group). **a**, Schematic of the experimental comparison for **b–e**, where the transcriptome of old UT-treated mice was compared to that of young UT-treated animals. **b**, UMAP visualization of small intestinal cell types generated by 10X chromium protocol. Color scale indicates difference in localized cellular density between UT-treated old and young mice. **c**, Fraction of cells for each of the different cell types shown in **b** in UT-treated old and young mice. **d**, UMAP visualization of small intestinal cell types generated by 10X chromium protocol. Color scale indicates log2FC differences in stemness signature score between UT-treated old and young mice. **e**, Split-violin plot indicates the expression level of five different stem-related genes in the stem cells from UT-treated old and young mice. Boxplots display median (center line) and interquartile range (box). **f**, Schematic of the experimental comparison for **g**–**j**, where the transcriptome of old m.uPAR-m.28z-treated mice was compared to that of old UT-treated animals. **g**, UMAP visualization of small intestinal cell types generated by 10X chromium protocol. Color scale indicates difference in localized cellular density between m.uPAR-m.28z- and UT-treated old mice. **h**, Fraction of cells for each of the different cell types shown in **g** in old mice treated with UT or m.uPAR-m.28z cells. **i**, UMAP visualization of small intestinal cell types generated by 10X chromium protocol. Color scale indicates log2FC differences in stemness signature score between m.uPAR-m.28z- and UT-treated old mice. **j**, Split-violin plot indicates the expression level of six different stem-related genes in the stem cells from old UT and m.uPAR-m.28z-treated mice. Boxplots display median (center line) and interquartile range (box). **k**, Experimental scheme for l-m: Young (3 months) and old (18 months) mice were treated with 0.5 × 10^6^ untransduced T cells (UT) or uPAR CAR T cells (m.uPAR-m.28z). Mice were harvested 8 weeks after infusion and organoids were generated from their intestinal crypts (*n* = 5 mice per group for UT young, m.uPAR-m.28z young and.uPAR-m.28z old and *n* = 4 mice for UT old, four to six replicates per mouse). **l**, Representative images of organoids at day 5. **m**, Number of organoids per field at day 4 (*n* = 5 mice per group for UT young, m.uPAR-m.28z young and.uPAR-m.28z old and *n* = 4 mice for UT old, four to six replicates per mouse). **n**, Experimental scheme for **o**: Young (3 months) and old (18 months) mice were treated with 0.5 × 10^6^ untransduced T cells (UT) or uPAR CAR T cells (m.uPAR-m.28z). Mice were harvested 8 weeks after infusion and organoids were generated from sorted EpCAM^+^ cells from their intestinal crypts. **o**, Number of organoids per field at day 4 (*n* = 4 mice per group, four to six replicates per mouse). Shown are results of one independent experiment (**a–j,n–o**) or two independent experiments (**k–m**). Significance was determined by two-tailed Wilcoxon rank-sum test (*P < 0.05,**P < 0.01, ***P < 0.001, ****P < 0.0001 (**e**,**j**) or two-tailed unpaired Student’s *t*-test (**m**,**o**). Data are mean ± s.e.m. (**m**,**o**). Illustration was created with Biorender.com (**a**,**f**,**k**,**n**). Source data

**Extended Data Fig. 5:**
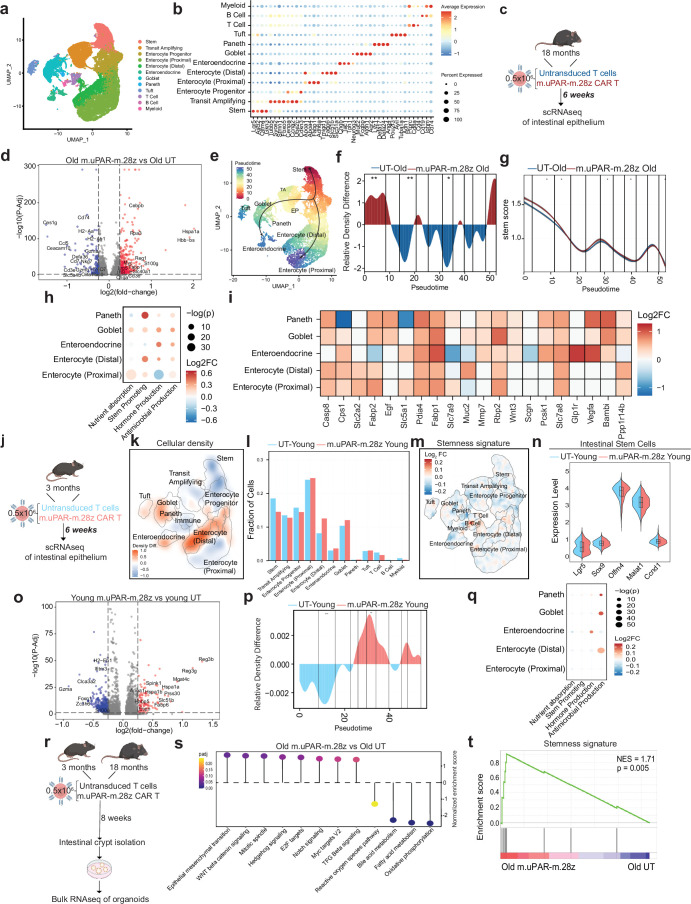
Therapeutic effect of uPAR-targeting CAR T cells on intestinal crypts. **a-q**, Young (3 months) and old (18 months) mice were treated with 0.5x10^6 untransduced T cells (UT) or uPAR CAR T cells (m.uPAR-m.28z). Mice were harvested 6 weeks after infusion and scRNAseq was performed from whole small intestine (n=4 mice per group pooled into 2 replicates) **a**, UMAP visualization of small intestinal cell types in young and old mice treated with 0.5x10^6 UT or m.uPAR-m.28z cells generated by 10X chromium protocol. Colors indicate the 12 different identified populations. **b**, Dot plot showing the 42 signature gene expressions across the 12 cellular clusters. The size of the dots represents the proportion of cells expressing a particular marker, and the color scale indicates the mean expression levels of the markers (log1p transformed). **c**, Schematic for d-i where the transcriptome of old m.uPAR-m.28z-treated mice was compared to that of old UT treated animals. **d**, Volcano plot of differentially expressed genes between old mice treated with UT or m.uPAR-m.28z cells. x-axis displays log2 fold change while y axis is the -log10 adjusted p-value as calculated by MAST. **e**, UMAP visualization from pseudotime trajectory analysis of cells from UT or m.uPAR-m.28z-treated old mice. Arrows highlight predicted trajectories. Color scale represents pseudotime. **f**, Density plot from pseudotime trajectory analysis demonstrating density differences along pseudotime of all cells between UT or m.uPAR-m.28z-treated old mice. **g**, Line plot showing the expression levels of stemness signature score in UT or m.uPAR-m.28z-treated old mice along the indicated pseudotime axis. **h**, Dot plot showing Log2 fold change in the functional scores for the different terms across cell types of old mice treated with UT or m.uPAR-m.28z cells. **i**, Heatmap representing log 2FC in gene expression between old m.uPAR-m.28z and UT treated mice from h. **j**, Schematic of k-q where the transcriptome of young mice treated with m.uPAR-m.28z CAR T cells or UT cells for 6 weeks was compared. **k**, UMAP visualization of small intestinal cell types generated by 10X chromium protocol. Color scale indicates difference in localized cellular density between m.uPAR-m.28z and UT treated young mice 6 weeks after treatment. **l**, Fraction of cells for each of the different cell types shown in (k) in young mice treated with UT or m.uPAR-m.28z cells for 6 weeks. **m**, UMAP visualization of small intestinal cell types generated by 10X chromium protocol. Color scale indicates log2FC differences in stemness signature score between m.uPAR-m.28z and UT treated young mice 6 weeks after treatment. **n**, Split-violin plot indicates the expression level of 5 stem-related genes in the stem cells from young UT and m.uPAR-m.28z-treated mice after 6 weeks. Boxplots display median (center line) and interquartile range (box).**o**, Volcano plot of differentially expressed genes between young mice treated with UT or m.uPAR-m.28z cells after. x-axis displays log2 fold change while y axis is the -log10 adjusted p-value as calculated by MAST. **p**, Density plot from pseudotime trajectory analysis demonstrating density differences along pseudotime of all cells between UT or m.uPAR-m.28z-treated young mice after 6 weeks. **q**, Dot plot showing Log2 fold change in the functional scores for the different terms across Paneth, goblet, enteroendocrine and enterocytes of young mice treated with UT or m.uPAR-m.28z cells after 6 weeks. **r**, Experimental scheme for s-t: Young (3 months) and old (18 months) mice were treated with 0.5x10^6 UT or uPAR CAR T cells. Mice were harvested 8 weeks after infusion and organoids were generated from their intestinal crypts. 5 days after, organoids were subjected to RNA sequencing. (n=4 mice per group pooled into 2 replicates). **s**, Pathway analysis comparing organoids from old m.uPAR-m.28z vs old UT treated mice. Enrichment assessed against Molecular Signature Database Hallmark 2025 gene sets. Color indicates adjusted p-value, y-axis represents normalized enrichment score. **t**, Gene set enrichment analysis of stemness signature in organoids from old m.uPAR-m.28z vs old UT treated mice. Results of 1 independent experiment (**a–t**). Two-tailed Fisher test (**f,p**). Two-tailed Wilcoxon rank-sum test *P<0.05,**P<0.01, ***P<0.001, ****P<0.0001 (**g-h,n,q**). MAST method: two-sided, p-values adjusted for multiple comparisons using the Benjamini-Hochberg method (**d,o**). Enrichment score in GSEA was calculated using a weighted Kolmogorov-Smirnov–like test. P values two-sided, non-parametric, permutation-based approach, with false discovery rate (FDR) correction applied for multiple comparisons. (**t**). Illustration created with Biorender.com (**c,j,r**).

To further explore the effects of uPAR CAR T treatment on ISCs in aged mice, we performed scRNA-seq of whole small intestine (duodenum, jejunum and ileum) in young (3 months) and old (20 months) mice 6 weeks after treatment with 0.5×10^6^ of uPAR CAR^+^ or UT cells (Fig. 4a–j and Extended Data Fig. 5a–q). We profiled 37,829 single cells and identified 12 different cell types, which were visualized using UMAP (Extended Data Fig. 5a,b).

In accordance with previous histological studies^13,39^ and our data (Fig. 2c and Extended Data Fig. 2p), the proportions of the different cell types varied with aging. Specifically, aged intestinal crypts presented a trend towards reduced abundance of ISCs (Fig. 4b,c). Importantly, these aged ISCs manifested a significant decrease in the expression levels of well-established stemness genes such as *Lgr4, Sox9, Olfm4* and *Malat1*, suggesting impaired stem cell activity with age (Fig. 4d,e)^4,13,39^. Interestingly, the intervention with uPAR CAR T cells in aged mice reversed this age-related decline in ISCs abundance and stemness gene expression (Fig. 4f–j and Extended Data Fig. 5d). Specifically, besides being present at higher proportions, ISCs from aged uPAR CAR T-treated mice were significantly enriched in stem cell signature genes compared to aged UT control mice (Fig. 4i,j). These observations were also supported by histological quantification of the number of ISC in the intestinal crypts (Fig. 2c and Extended Data Fig. 2p) as well as by pseudotime trajectory analysis that revealed an increase in the relative density difference and enrichment of the stemness score at the early pseudotime points in the aged uPAR CAR T treated mice over controls (Extended Data Fig. 5e–g).

To assess the regenerative potential of these ISCs we performed clonogenic organoid formation assays from epithelial crypts (Fig. 4k–m) as well as from sorted EpCAM^+^ cells from the small intestines of young and old, uPAR CAR T or UT-control treated mice 6 weeks after infusion (Fig. 4n,o). Congruent with previous reports^4,5,7^, crypts from old mice generated significantly fewer organoids than those from young animals (Fig. 4l,m,o). However, *in vivo* treatment with uPAR CAR T cells rescued the ability of both aged crypts and sorted EpCAM^+^ cells to efficiently generate organoids (Fig. 4l,m,o). In addition, the organoids generated from the crypts of aged in vivo uPAR CAR T treated mice presented increased expression of stemness, proliferation and WNT pathway signatures (Extended Data Fig. 5r–t). Notably, we also observed similar results in organoid formation assays from the epithelial crypts of aged D + Q-treated mice compared to aged controls (Extended Data Fig. 3f,g). Beyond ISCs, aging results in deficits in the functions of mature epithelial cell types such as Paneth, goblet, enteroendocrine cells and enterocytes^4,40,41^. Compared to UT controls, in vivo treatment with uPAR CAR T cells in old mice elicited gene expression changes in these mature epithelial cell types that potentially correlate with increased functional fitness (Extended Data Fig. 5h,i).

**Fig. 5:**
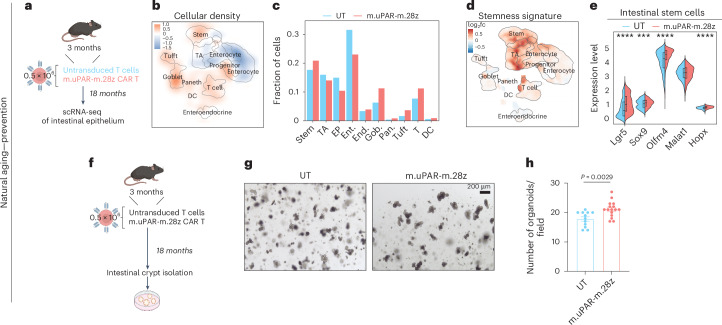
Prophylactic uPAR CAR T cells rejuvenate ISCs. **a**, Experimental scheme for **q**–**t**: Young (3 months) mice were treated with 0.5 × 10^6^ untransduced T cells (UT) or uPAR CAR T cells (m.uPAR-m.28z). Mice were harvested 15 months after infusion at the age of 18 months, and scRNA-seq was performed from whole small intestine: duodenum, jejunum and ileum (*n* = 1 per group). **b**, UMAP visualization of small intestinal cell types generated by 10X chromium protocol. Color scale indicates difference in localized cellular density between m.uPAR-m.28z and UT treated mice. **c**, Fraction of cells for each of the different cell types shown in **q** in mice treated with UT or m.uPAR-m.28z cells. **d**, UMAP visualization of small intestinal cell types generated by 10X chromium protocol. Color scale indicates log2FC differences in stemness signature score between m.uPAR-m.28z- and UT-treated mice. **e**, Split-violin plot indicates the expression level of five different stem-related genes in the stem cells from old UT- and m.uPAR-m.28z-treated mice. Boxplots display median (center line) and interquartile range (box). **f**, Experimental scheme for **v**–**w**: Young (3 months) mice were treated with 0.5 × 10^6^ untransduced T cells (UT) or uPAR CAR T cells (m.uPAR-m.28z). Mice were harvested 15 months after infusion at the age of 18 months and organoids were generated from their intestinal crypts (*n* = 3 mice per group for UT and *n* = 4 mice for m.uPAR-m.28z, four replicates per mouse). **g**, Representative images of organoids at day 4. **h**, Number of organoids per field at day 4 (*n* = 3 mice per group for UT and *n* = 4 mice for m.uPAR-m.28z, four replicates per mouse). Shown are results of one independent experiment (**a–h**). Significance was determined by two-tailed Wilcoxon rank-sum test (*P < 0.05,**P < 0.01, ***P < 0.001, ****P < 0.0001) (**e**). Data are mean ± s.e.m. (**h**). Significance was determined by two-tailed unpaired Student’s *t*-test (**h**). Illustration was created with Biorender.com (**a**,**f**). Source data

**Extended Data Fig. 6:**
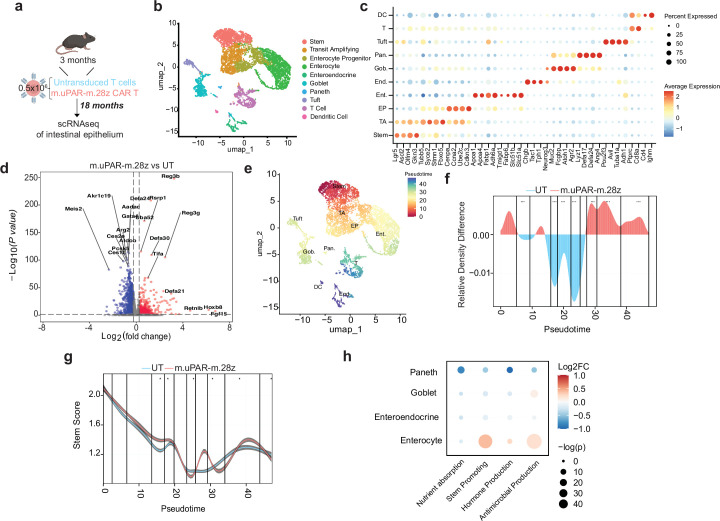
Prophylactic effect of uPAR-targeting CAR T cells on intestinal crypts. **a**, Experimental scheme for b-h: Young (3 months) mice were treated with 0.5x10^6 untransduced T cells (UT) or uPAR CAR T cells (m.uPAR-m.28z). Mice were harvested 15 months after infusion at the age of 18 months and scRNAseq was performed from whole small intestine: duodenum, jejunum and ileum. (n=1 per group). **b**, UMAP visualization of small intestinal cell types generated by 10X chromium protocol. Colors indicate the 10 different identified populations. **c**, Dot plot showing the 40 signature gene expressions across the 10 cellular clusters. The size of the dots represents the proportion of cells expressing a particular marker, and the color scale indicates the mean expression levels of the markers (log1p transformed). **d**, Volcano plot of differentially expressed genes between young mice treated with UT or m.uPAR-m.28z cells 15 months after treatment. x-axis displays log2 fold change while y axis is the -log10 adjusted p-value as calculated by MAST. **e**, UMAP visualization from pseudotime trajectory analysis of cells from UT or m.uPAR-m.28z treated young mice 15 months after treatment. Arrows highlight predicted trajectories within cell clusters. Color scale represents pseudotime. **f**, Density plot from pseudotime trajectory analysis demonstrating density differences along pseudotime of all cells between UT or m.uPAR-m.28z treated young mice 15 months after treatment. **g**, Line plot showing the expression levels of stemness signature score in UT or m.uPAR-m.28z treated young mice 15 months after treatment along the indicated pseudotime axis. **h**, Dot plot showing Log2 fold change in the functional scores for the different terms across Paneth, goblet, enteroendocrine and enterocytes of young mice treated with UT or m.uPAR-m.28z cells 15 months after treatment. Results of 1 independent experiment (**a-h**). Two-tailed Fisher test (**f**). Two-tailed Wilcoxon rank-sum test *P<0.05,**P<0.01, ***P<0.001, ****P<0.0001 (**g-h**). MAST method: two-sided, p-values adjusted for multiple comparisons using the Benjamini-Hochberg method (**d**). Illustration created with Biorender.com (**a**).

To understand how treatment with uPAR CAR T cells impacted young mice, we first profiled their intestinal crypts 6 weeks after cell infusion but did not observe notable effects on either cell composition or the expression of key stemness genes (Extended Data Fig. 5j–q). However, when we performed scRNA-seq 15 months later (when the animals were now 18 months old) (Fig. 5a–e and Extended Data Fig. 6) we found a trend toward increased ISC abundance (which was validated at the histological level (Fig. 3c and Extended Data Fig. 2w) and by pseudotime trajectory analysis (Extended Data Fig. 6e,f)) and increased expression of the stemness gene expression program in ISCs from uPAR CAR T-treated mice compared to UT controls (Fig. 5d,e). Accordingly, the intestinal crypts from prophylactically treated aged mice were able to form significantly higher numbers of organoids than those from UT-treated animals (Fig. 5f–h).

Collectively, these data indicate that removal of uPAR^+^ cells (either therapeutically in aged mice or prophylactically throughout life) enhances ISC activity and regeneration potential upon aging.

### Prophylactic or therapeutic treatment with uPAR CAR T cells improves age-associated intestinal inflammation and dysbiosis

**Fig. 6:**
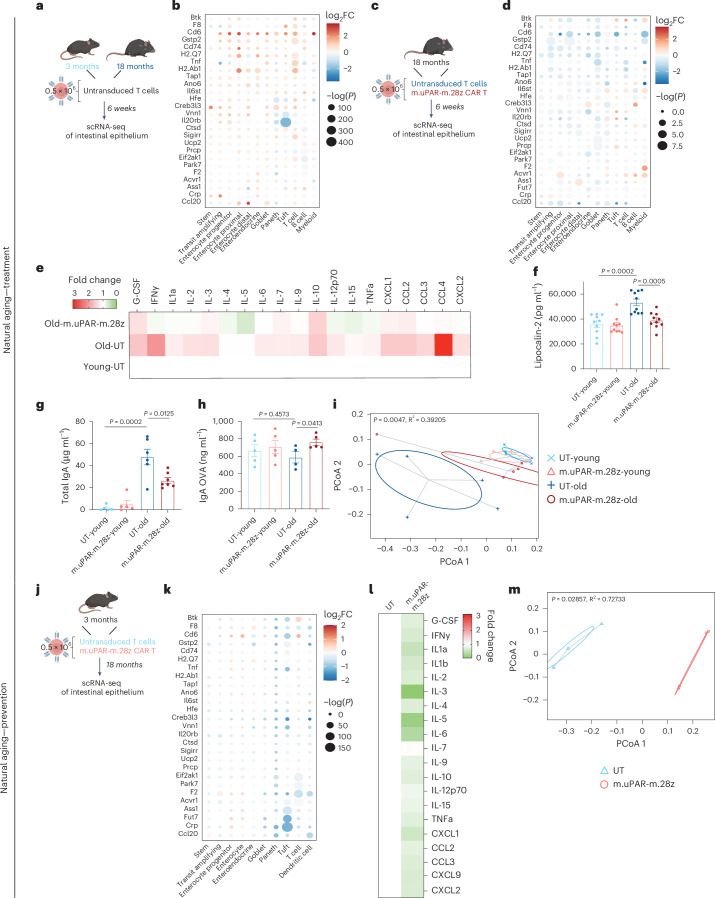
uPAR^+^ cells drive chronic age-related intestinal inflammation. **a-i**, Young (3 months) and old (18 months) mice were treated with 0.5 × 10^6^ untransduced T cells (UT) or uPAR CAR T cells (m.uPAR-m.28z). Mice were harvested 6 weeks after infusion and (for **a–d**) scRNA-seq was performed from whole small intestine: duodenum, jejunum and ileum (*n* = 4 mice per group) **a**, Schematic of the experimental comparison for b where the transcriptome of old UT-treated mice was compared to that of young UT treated animals (*n* = 4 mice per group pooled into 2 replicates per group). **b**, Dot plot depicting differential expression of various immunomodulatory genes for different cell types in old UT mice versus young UT infused mice 6 weeks after infusion. Color scale represents average log2FC and size scale represents the degree of significance (*n* = 4 mice per group pooled into two replicates per group). **c**, Schematic of the experimental comparison for **d**, where the transcriptome of old m.uPAR-m.28z-treated mice was compared to that of old UT-treated animals (*n* = 4 mice per group pooled into two replicates per group). **d**, Dot plot depicting differential expression of various immunomodulatory genes for different cell types in old uPAR CAR T-treated mice versus old UT-infused mice 6 weeks after infusion. Color scale represents average log2FC, and size scale represents the degree of significance (*n* = 4 mice per group pooled into two replicates per group). **e**, Heatmap depicting the fold change in the protein levels of proinflammatory cytokines and chemokines in the intestinal epithelium 20 days after cell infusion (*n* = 4 mice per group). **f**, Plasma levels of lipocalin-2 20 days after cell infusion (*n* = 10 mice per group). **g**, Serum levels of total unspecific IgA in young and old mice 20 days after cell infusion (young UT *n* = 5, young m.uPAR-m.28z *n* = 5, old UT *n* = 6 mice, old m.uPAR-m.28z *n* = 7 mice). **h**, Young (3 m) and old (20 m) mice were infused with 0.5 × 10^6^ UT or m.uPAR-m.28z CAR T cells. 20 days after infusion, mice were immunized by oral gavage with OVA and cholera toxin on three occasions separated by 7 days. Serum was collected on day 21, and levels of specific anti-OVA IgA were determined by ELISA. (Young UT *n* = 5, young.m.uPAR-m.28z *n* = 5, old UT *n* = 4 mice, old m.uPAR-m.28z *n* = 5 mice). **i**, Principal coordinate analysis (PCoA) of the microbial composition in fecal samples of young (3 months) and old (20 months) mice 20 days after infusion with 0.5 × 10^6^ UT or m.uPAR-m.28z CAR T cells (*n* = 5 mice per group). **j–m**, Young (3 months) mice were treated with 0.5 × 10^6^ untransduced T cells (UT) or uPAR CAR T cells (m.uPAR-m.28z). Mice were harvested 15 months after infusion at the age of 18 months, and for **j–k**, scRNA-seq was performed from whole small intestine: duodenum, jejunum and ileum. **j**, Schematic of the experimental comparison for **k**, where the transcriptome of m.uPAR-m.28z-treated mice was compared to that of UT-treated animals (*n* = 1 per group). **k**, Dot plot depicting differential expression of various immunomodulatory genes for different cell types in uPAR CAR T-treated mice versus UT infused mice. Color scale represents average log2FC, and size scale represents the degree of significance (*n* = 1 per group). **l**, Heatmap depicting the fold change in the protein levels of proinflammatory cytokines and chemokines 15 months after cell infusion (*n* = 3 for UT and *n* = 4 for m.uPAR-m.28z). **m**, PCoA of the microbial composition in fecal samples 15 months after cell infusion (*n* = 3 for UT and *n* = 4 for m.uPAR-m.28z). Shown are results of one independent experiment (**a–m**). Data are mean ± s.e.m. (**f–h**). Significance was determined by two-tailed unpaired Student’s *t*-test (**b**,**d**,**f–h**,**k**) or two-tailed PERMANOVA (**i**,**m**). Illustration was created with Biorender.com (**a**,**c**,**j**). Source data

In our scRNA-seq data, we observed a significant upregulation in the expression of genes related to inflammation in the small intestines of aged mice compared to young counterparts (Fig. 6a,b), which could potentially reflect inflammaging and the proinflammatory SASP^14,41^. Treatment of old mice with uPAR CAR T cells significantly abrogated the expression of these inflammatory response genes (Fig. 6c,d). Beyond changes in gene expression, aged uPAR CAR T-treated mice also presented decreased protein expression of proinflammatory cytokines and chemokines (such as IFNy, IL6, CXCL1 or CCL4) in their small intestinal epithelium, as measured by cytokine array (Fig. 6e), as well as decreased levels of circulating intestinal inflammatory markers such as lipocalin-2 (Fig. 6f).

**Extended Data Fig. 7:**
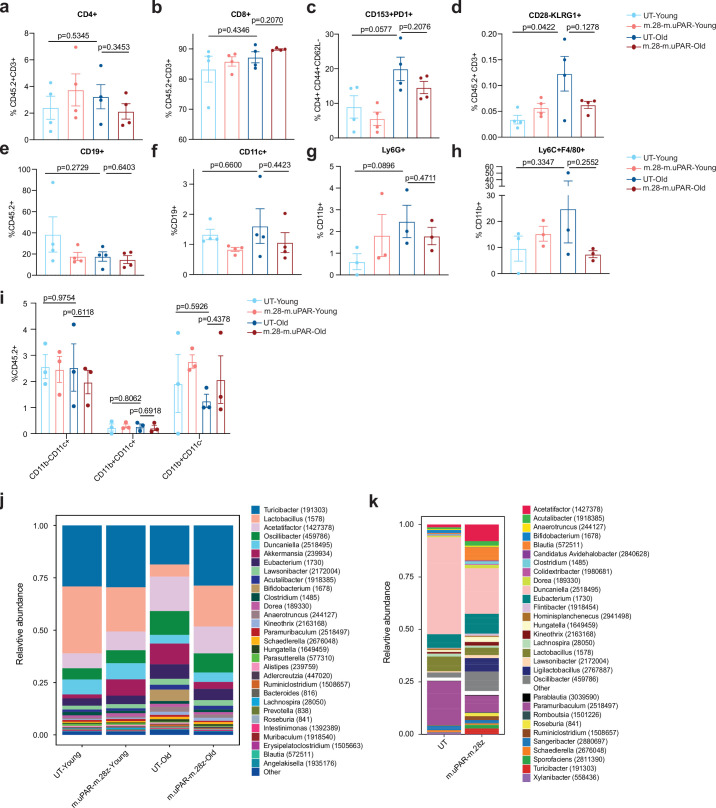
Senolytic CAR T cells abrogate features of intestinal inflammaging. **a-i**, Young (3 months) and old (18 months) mice were treated with 0.5x10^6 untransduced T cells (UT) or uPAR CAR T cells (m.uPAR-m.28z). Mice were harvested 6 weeks after infusion and flow cytometry was performed on intestinal epithelium. **a**, Percentage of CD4 positive cells from CD45.2 and CD3 double positive cells in the intestinal crypts. (n=4 per group). **b**, Percentage of CD8 positive cells from CD45.2 and CD3 double positive cells in the intestinal crypts. (n=4 per group). **c**, Percentage of senescent endogenous T cells (CD153+PD1+) from CD4+CD44+CD62L- T cells in the intestinal crypts (n=4 per group). **d**, Percentage of senescent endogenous T cells (CD28- KLRG1+) from total CD45.2+ CD3+ cells in the intestinal crypts (n=4 per group). **e**, Percentage of CD19 positive cells from CD45.2 positive cells in the intestinal crypts. (n=4 per group). **f**, Percentage of CD11c positive cells from CD19 positive cells in the intestinal crypts. (n=4 per group). **g**, Percentage of Ly6G positive cells from CD11b positive cells in the intestinal crypts. (n=3 per group). **h**, Percentage of Ly6C, F4/80 double positive cells from CD11b positive cells in the intestinal crypts. (n=3 per group). **i**, Percentage of CD11b, CD11c and double positive cells from CD45.2 positive cells in the intestinal crypts. (n=3 per group). **j**, Relative abundance of microbial genus from Fig. 4i as determined by metagenomics analysis in each treatment condition (n=5 per group). **k**, Relative abundance of microbial genus from Fig. 4m as determined by metagenomics analysis in each treatment condition (n=3 for UT, n=4 for m.uPAR-m.28z). Results of 1 independent experiment (**a-k**). Data are mean ± s.e.m. (**a-i**). Two-tailed unpaired Student’s t-test (**a-i**). Source data

Functionally, the reduction in intestinal inflammation following uPAR CAR T treatment correlated with decreased proportions in the aged intestinal epithelium of immune cells displaying markers associated with immunosenescence such as CD28^−^ KLRG1^+^ T cells (Extended Data Fig. 7a–i). In addition, aged animals treated with uPAR CAR T cells presented decreased levels of nonspecific IgA, a marker of gut mucosal inflammaging^42^ (Fig. 6g), and mounted stronger antigen-specific responses to mucosal vaccines (Fig. 6h). Together, this suggests that ablation of uPAR^+^ cells enhances the function of the endogenous mucosal immune system in aged mice.

Interestingly, we also observed that the microbiome composition of aged uPAR CAR T-treated mice was significantly more similar to that of younger animals (Fig. 6i and Extended Data Fig. 7j). Moreover, although less pronounced, the microbiome of mice treated with D + Q was also more similar to that of young animals (Extended Data Fig. 3h,i)^43^. Comparison of the changes induced in fecal microbial composition by uPAR CAR T cells with those induced by D + Q revealed similar trends such as the increased abundance in the genus of *Lactobacillus*, but also differences such as increase in *Turicibacter* with senolytic CAR T cells but a decrease in the abundance of this genus with D + Q.

Prophylactic treatment with uPAR CAR T cells had similar effects on reducing intestinal inflammation and changes to the microbiome composition once the mice reached 18 months (Fig. 6j–m). Thus, prophylactically treated mice presented decreased expression of inflammatory genes as well as decreased protein levels of proinflammatory cytokines and chemokines (such as IFNy, IL6 or CXCL1) in their intestinal epithelium (Fig. 6k,l). In addition, the microbiome composition of uPAR CAR T prophylactically treated mice was also significantly different from that of controls, presenting similarities to that of mice treated with uPAR CAR T cells at 18 months old (such as increased abundance of *Turicibacter*) but also differences (such as decreased *Lactobacillus*) (Fig. 6m and Extended Data Fig. 7k).

Overall, these results suggest that uPAR^+^ cells play a key role in chronic age-related intestinal inflammation and that their elimination, either therapeutically or prophylactically, with uPAR CAR T cells can significantly ameliorate intestinal inflammaging and modify the microbiome composition.

### Direct targeting of epithelial, but not immune, uPAR^+^ cell populations is sufficient to improve the regenerative capacity of aged ISCs

**Fig. 7:**
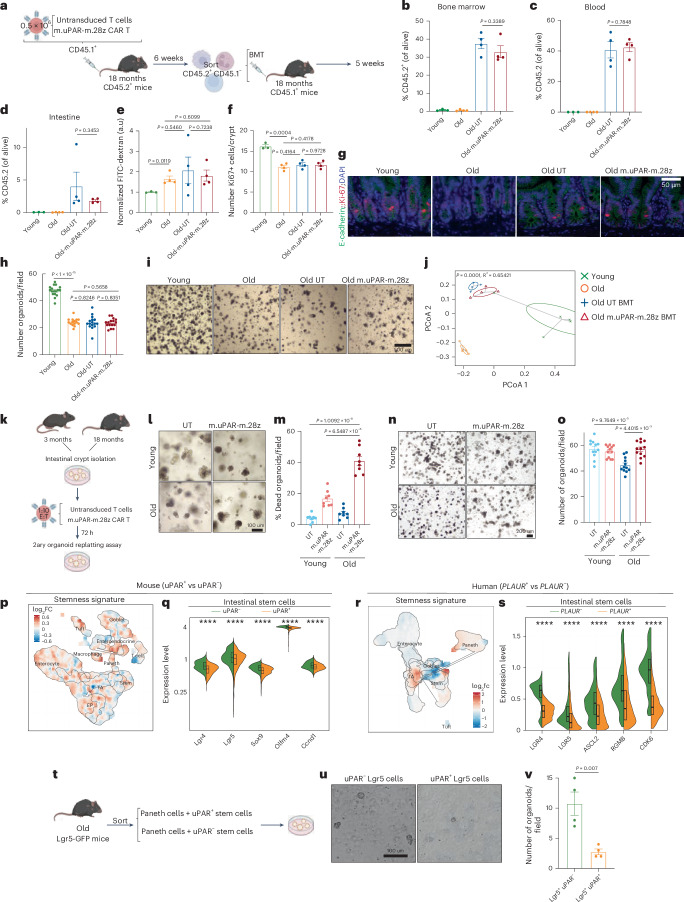
Targeting uPAR^+^ epithelial but not immune cells is sufficient to improve regeneration of aged intestinal crypts. **a**, Experimental scheme for **a–j**: 0.5 × 10^6^ UT or m.uPAR-m.28z cells generated from CD45.1^+^ mice were infused into 18-month-old CD45.2^+^ mice. Six weeks later, 0.5 × 10^6^ CD45.2^+^CD45.1^−^ cells were isolated from their bone marrow and transplanted into 18-month-old CD45.1^+^ mice that had been preconditioned with busulfan (30 mg kg^−1^ in 3 consecutive days). Transplanted mice, alongside controls young (3 months) and old (18 months) animals were euthanized 5 weeks later. **b**, Percentage of CD45.2^+^ cells in the bone marrow (*n* = 3 mice for young, *n* = 4 mice for the other groups). **c**, Percentage of CD45.2^+^ cells in peripheral blood (*n* = 3 mice for young, *n* = 4 mice for the other groups. **d**, Percentage of CD45.2^+^ cells in intestinal epithelium (*n* = 3 mice for young, *n* = 4 mice for the other groups). **e**, Normalized plasma levels of FITC-Dextran 4 h after oral gavage (*n* = 3 mice for young, *n* = 4 mice for the other groups). **f**, Quantification of the number of Ki-67^+^ cells per intestinal crypt in the proximal jejunum (*n* = 3 mice for young, *n* = 4 mice for mice for the other groups). **g**, Representative immunofluorescence staining of E-Cadherin (green), Ki-67 (red) and DAPI (blue) in the proximal jejunum (*n* = 3 mice for young, *n* = 4 mice for the other groups). **h**, Number of organoids per field at day 4 (*n* = 4 mice per group, four to five replicates per mouse). **i**, Representative images of organoids at day 4. **j**, PCoA of the microbial composition in fecal samples (*n* = 5 mice for young, *n* = 5 mice for old, *n* = 4 mice for the other groups). **k**, Experimental scheme for **l**–**o**: Intestinal crypts from *n* = 4 young (3 months) and *n* = 4 old (20 months) mice were isolated and seeded to form organoids together with either UT or m.uPAR-m28z cells at 1:10 effector/target ratio. Then, 72 h later, equal numbers of secondary organoids were generated per dissociated crypt-derived primary organoids. **l**, Representative images of organoids after co-culture with UT or m.uPAR-m28z cells for 72 h. **m**, Quantification of the percentage of dead organoids per field 72 h after co-culture between organoids and UT or m.uPAR-m28z cells (*n* = 4 young mice were pooled to generate eight replicates per condition and *n* = 4 old mice were pooled to generate eight replicates per condition. **n**, Representative images of secondary organoids from young and old in vitro UT or m.uPAR-m28z CAR T-treated primary organoids at day 4. **o**, Quantification of number of secondary organoids on day 4 (*n* = 12 replicates per group). **p**, UMAP visualization of murine small intestinal cell types generated by 10X chromium protocol. Color scale indicates log2FC differences in stemness signature score between mouse uPAR^+^ and uPAR^−^ cells (*n* = 4 mice per group pooled into two replicates per group). **q**, Split-violin plot indicates the expression level of five different stem-related genes in mouse uPAR^+^ or uPAR^−^ stem cells (*n* = 4 mice per group pooled into two replicates per group). Boxplots display median (center line) and interquartile range (box). **r**, UMAP visualization of human non-immune small intestinal cell types generated by 10X chromium protocol^33^ (*n* = 1 per group). Color scale indicates log2FC differences in stemness signature score between human *PLAUR*^*+*^ and *PLAUR*^−^ cells. **s**, Split-violin plot indicates the expression level of 5 different stem-related genes in human *PLAUR*^*+*^ or *PLAUR*^−^ stem cells from duodenum, jejunum and ileum^33^(*n* = 1 per group). Boxplots display median (center line) and interquartile range (box). **t**, Experimental scheme for u–v: 50,000 uPAR^+^ or uPAR^−^ Lgr5^+^ cells and Lgr5^−^CD24^+^ cells were sorted from the intestinal epithelium of aged (25 months old) Lgr5-GFP mice and seeded to form organoids (*n* = 3 mice). **u**, Representative organoid images at day 8 (four replicates per group). **v**, Quantification of number of organoids on day 8 (four replicates per group). Shown are results of one independent experiment (**a–j**,**p–v**) or two independent experiments (**k–o**). Significance was determined by two-tailed Wilcoxon rank-sum test (*P< 0.05,**P < 0.01, ***P < 0.001, ****P < 0.0001) (**q**,**s**). Data are mean ± s.e.m. (**b–f**,**h**,**m**,**o**,**v**). Significance was determined by two-tailed unpaired Student’s *t*-test (**b–f**,**h**,**m**,**o**,**v**) or two-tailed PERMANOVA (**j**). Illustration was created with Biorender.com (**a**,**k**,**t**). Source data

**Extended Data Fig. 8:**
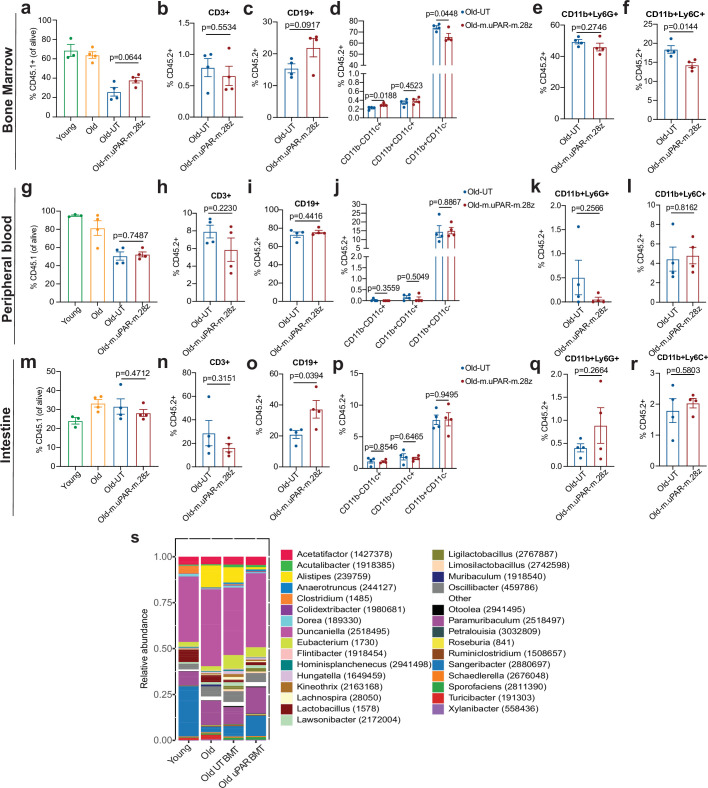
Effects of transplanting the immune system from aged uPAR CAR T treated mice. **a–s:** 0.5x10^6^ UT or m.uPAR-m.28z cells generated from CD45.1^+^ mice were infused into 18 months old CD45.2^+^ mice. 6 weeks later, 0.5x10^6^ CD45.2^+^CD45.1^−^ were isolated from the bone marrow of these mice and transplanted into aged (18 months old) CD45.1^+^ mice that had been preconditioned with busulfan (30mg/kg in 3 consecutive days). Transplanted mice, alongside controls young (3 months) and old (18 months old) animals were euthanized 5 weeks later and employed for experiments. **a**, Percentage of CD45.1 positive cells in the bone marrow (n=3 mice for young, n=4 mice for old, n=4 mice for old mice transplanted with the immune system of aged UT treated mice, n=4 for old mice transplanted with the immune system of aged m.uPAR-m.28z treated mice). **b**, Percentage of CD3 positive cells from CD45.2 positive cells in the bone marrow (n=4 per group). **c**, Percentage of CD19 positive cells from CD45.2 positive cells in the bone marrow (n=4 per group). **d**, Percentage of CD11b positive, CD11c positive or both CD11b and CD11c positive cells from CD45.2 positive cells in the bone marrow (n=4 per group). **e**, Percentage of double CD11b positive and Ly6G positive cells from CD45.2 positive cells in the bone marrow (n=4 per group). **f**, Percentage of double CD11b positive and Ly6C positive cells from CD45.2 positive cells in the bone marrow (n=4 per group). **g**, Percentage of CD45.1 positive cells in the peripheral blood (n=3 mice for young, n=4 mice for old, n=4 mice for old mice transplanted with the immune system of aged UT treated mice, n=4 for old mice transplanted with the immune system of aged m.uPAR-m.28z treated mice). **h**, Percentage of CD3 positive cells from CD45.2 positive cells in the peripheral blood (n=4 per group). **i**, Percentage of CD19 positive cells from CD45.2 positive cells in the peripheral blood (n=4 per group). **j**, Percentage of CD11b positive, CD11c positive or both CD11b and CD11c positive cells from CD45.2 positive cells in the peripheral blood (n=4 per group). **k**, Percentage of double CD11b positive and Ly6G positive cells from CD45.2 positive cells in the peripheral blood (n=4 per group). **l**, Percentage of double CD11b positive and Ly6C positive cells from CD45.2 positive cells in the peripheral blood (n=4 per group). **m**, Percentage of CD45.1 positive cells in the intestinal epithelium (n=3 mice for young, n=4 mice for old, n=4 mice for old mice transplanted with the immune system of aged UT treated mice, n=4 for old mice transplanted with the immune system of aged m.uPAR-m.28z treated mice). **n**, Percentage of CD3 positive cells from CD45.2 positive cells in the intestinal epithelium (n=4 per group). **o**, Percentage of CD19 positive cells from CD45.2 positive cells in the intestinal epithelium (n=4 per group). **p**, Percentage of CD11b positive, CD11c positive or both CD11b and CD11c positive cells from CD45.2 positive cells in the intestinal epithelium (n=4 per group). **q**, Percentage of double CD11b positive and Ly6G positive cells from CD45.2 positive cells in the intestinal epithelium (n=4 per group). **r**, Percentage of double CD11b positive and Ly6C positive cells from CD45.2 positive cells in the intestinal epithelium (n=4 per group). **s**, Relative abundance of microbial genus as determined by metagenomics analysis in each condition (n=5 mice for young, n=5 mice for old, n=4 mice for old mice transplanted with the immune system of aged UT treated mice, n=4 for old mice transplanted with the immune system of aged m.uPAR-m.28z treated mice). Results of 1 independent experiment (**a–s**). Data are mean ± s.e.m. (**a-r**). Two-tailed unpaired Student’s t-test (**a-r**). Source data

**Extended Data Fig. 9:**
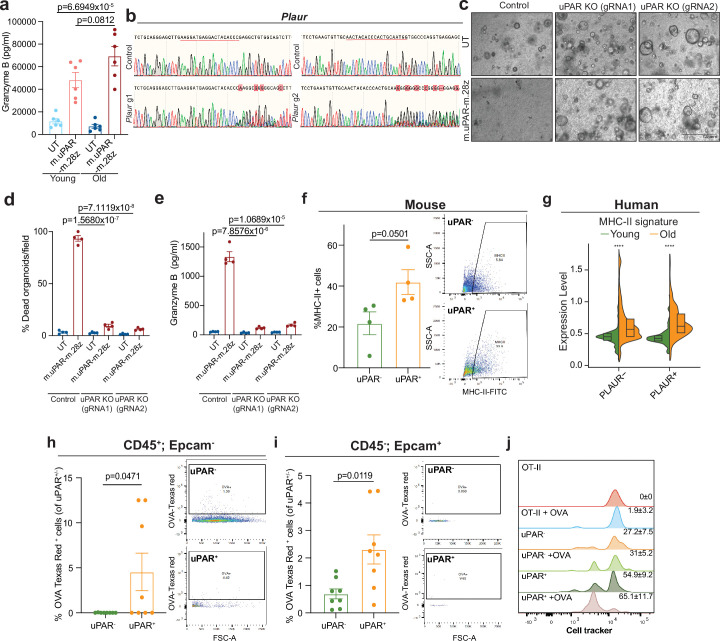
Characteristics of uPAR+ epithelial cells. **a**, Intestinal crypts from n=4 young (3 months) and n=4 old (20 months old) mice were isolated and seeded to form organoids together with either UT or m.uPAR-m28z cells at 1:10 effector:target ratio. Levels of granzyme B 72h after co-culture between organoids and UT or m.uPAR-m28z cells (n=6 replicates per group). **b-e**, Aged murine intestinal organoids were edited by CRISPR-Cas9-sgRNAs targeting *Plaur*. **b**, Sanger sequencing confirming editing of the *Plaur* locus. **c-e:** organoids were cultured together with either UT or m.uPAR-m28z cells at 1:10 effector:target ratio for 72 h (n=4 replicates per group). **c**, Representative images of organoids after co-culture with UT or m.uPAR-m28z cells for 72 h. **d**, Quantification of the percentage of dead organoids per field 72h after co-culture between organoids and UT or m.uPAR-m28z cells (n=4 replicates per group). **e**, Levels of granzyme B 72h after co-culture between organoids and UT or m.uPAR-m28z cells (n=4 replicates per group). **f**, Percentage of cells expressing surface MHC-II as determined by flow cytometry on isolated intestinal crypts from old (18 months old) mice. (n=4 per group). **g**, Split-violin plot indicates the expression level of MHC-II signature score in non-immune *PLAUR-* or *PLAUR+* cells from the duodenum, jejunum and ileum of young (25–30 years old) and old (65–70 years old) subjects^33^. Boxplots display median (center line) and interquartile range (box) (n=1 per group).**h-i**, 18 months old mice were administered 1mg/ml ovalbumin conjugated to Texas red by oral gavage. Intestinal crypts were isolated and dissociated 1h after administration. **h**, Graph depicting percentage of CD45^+^ EpCAM^−^ uPAR^−^ or uPAR^+^ cells positive for Texas red. (n=8 mice per group). **i**, Graph depicting percentage of CD45^−^ EpCAM^+^ uPAR^−^ or uPAR^+^ cells positive for Texas red. (n=8 mice per group). **j**, CD45^−^ EpCAM^+^ uPAR^+^ cells induce CD4^+^ T cell proliferation *in vitro*. Representative FACS histograms of cell trace violet-labeled CD4^+^ T cells from OT-II mice that were cultured alone or with CD45^−^ EpCAM^+^ uPAR^−^ or CD45^−^ EpCAM^+^ uPAR^+^ cells from 18 month old mice with or without 15ug/ml of OVA323-339. Values shown are mean ± s.d. (n=3 for T cells alone and T cells alone with OVA; n=4 mice per group for the other groups). Results of 1 independent experiment (**a-g,j**). Results of 2 independent experiment (**h-i**). Data are mean ± s.e.m. (**a,d-f,h-i**). Data are mean ± s.d (**j**). Two-tailed unpaired Student’s t-test (**a,d-f,h-i**). Two-tailed Wilcoxon rank-sum test *P<0.05,**P<0.01, ***P<0.001, ****P<0.0001 (**g**). Source data

To gain a better understanding of which cell types mediate the detrimental effects on intestinal homeostasis during aging, we performed experiments designed to assay the effects of targeting only immune or only epithelial cells with uPAR CAR T cells (Fig. 7a–v and Extended Data Figs. 8 and 9a).

To investigate the impact on intestinal homeostasis of targeting only immune cells, we performed a transplant experiment in which aged (18 months old) CD45.2 mice were infused with 0.5 × 10^6^ UT or uPAR CAR T cells generated from CD45.1 mice. Six weeks after administration, their whole bone marrow (excluding the infused T cells) was sorted as CD45.2^+^ CD45.1^−^ and transplanted into aged CD45.1 mice (Fig. 7a). Five weeks after the transplant, engraftment rate was comparable between aged mice that received hematopoietic cells from aged UT-treated donors and those that received cells from aged uPAR CAR T-treated donors (Fig. 7b–d). Transplanted cells were detectable in the bone marrow, blood and also in the intestine, indicating successful hematopoietic reconstitution and partial replacement of tissue-resident immune cells (Fig. 7b–d and Extended Data Fig. 8a–r). However, this was not sufficient to improve the aging-induced increased intestinal permeability (Fig. 7e), decreased proliferation in the crypts (Fig. 7f,g), decreased organoid-forming ability (Fig. 5h,i) or dysbiosis (Fig. 7j and Extended Data Fig. 8s). Although not all tissue-resident immune cells are replaced by the bone marrow transplant, these results suggest that targeting only the immune compartment is insufficient to recapitulate the observed improvements with uPAR CAR T cell therapy.

As transplantation experiments are not feasible on the intestinal epithelial compartment, we instead co-cultured crypts from young (3 months) and old (20 months) mice with either UT cells or uPAR CAR T cells to directly target the uPAR^+^ epithelial compartment in vitro (Fig. 7k–m and Extended Data Fig. 9a). As expected, uPAR CAR T cells preferentially targeted old crypts (Fig. 7l,m and Extended Data Fig. 9a) and did so in a specific manner, sparing uPAR knockout (KO) aged organoids (Extended Data Fig. 9b–e). Notably, dissociated single cells from uPAR CAR T-treated old organoids gave rise to significantly more organoids in secondary subcultures compared to UT old-treated controls, suggesting that direct elimination of uPAR^+^ epithelial cells was sufficient to enhance the regenerative capacity of aged crypts (Fig. 7n,o).

To understand whether the increased regenerative capacity was due to direct effects of uPAR CAR T cells on the ISCs, we compared the stemness gene signature of aged uPAR^+^ and uPAR^−^ ISCs (Fig. 7p,q). Notably, uPAR^+^ ISCs had significantly lower expression of key stemness genes such as *Lgr4, Lgr5, Sox9, Olfm4* and *Ccnd1* than uPAR^−^ ISCs (Fig. 7p,q). Similarly, analysis of *PLAUR* gene expression in aged human ISCs showed that *PLAUR*^*+*^ ISCs have reduced expression levels of genes involved in stem cell activity compared to *PLAUR*^−^ ISCs (Fig. 7r,s). To assay whether uPAR^+^ ISCs had decreased regenerative potential, we sorted Lgr5^+^ uPAR^+^/uPAR^−^ cells from aged Lgr5-GFP mice and tested their organoid-forming ability (Fig. 7t). Indeed, we found that Lgr5^+^ uPAR^+^ ISCs formed significantly fewer organoids compared with Lgr5^+^ uPAR^−^ ISCs (Fig. 7u,v). Overall, our results suggest that surface uPAR expression identifies dysfunctional ISCs and that their direct targeting through uPAR CAR T cells is sufficient to improve the regenerative capacity of aged intestinal crypts.

Together, our data show that in vivo treatment with uPAR CAR T cells improves ISC activity and significantly decreases intestinal inflammation during aging (Figs. 4i–o, 5d–h and 6d,e,k,l). Curiously, some of the most differentially expressed inflammatory genes related to those encoding MHC-II molecules (such as *H2-Ab1*) (Fig. 6b,d,k). We and others have recently shown that epithelial MHC-II expression in young animals mediates immune cell-ISC crosstalk in the intestinal epithelium, influencing inflammation, response to infection and anti-tumor immunity^44,45^. Intrigued by the downregulation of MHC-II expression after uPAR CAR T treatment, we examined whether uPAR^+^ cells themselves expressed MHC-II. Indeed, we found that MHC-II expression was significantly increased on aged intestinal uPAR^+^ epithelial cells in both mice and humans (Extended Data Fig. 9f,g).

To explore whether naturally occurring uPAR^+^ cells in the intestine could potentially uptake antigens, we orally administered ovalbumin conjugated to Texas red dye to aged animals and examined the percentage of Texas red-positive cells in the intestines of these mice 1 h after administration. Interestingly, we found that uPAR^+^ cells were able to uptake antigen (Extended Data Fig. 9h,i). To further study whether epithelial uPAR^+^ cells could not only uptake antigen but also present it on MHC-II molecules and activate CD4 T cell responses, we sorted CD45^−^, EpCAM^+^, uPAR^+^ or uPAR^−^ cells and co-cultured them with ovalbumin 323–339 peptide and OT-II cells (which are specific for OVA323–339 presented by MHC-II molecules). Indeed, we observed that uPAR^+^ epithelial cells were able to stimulate OT-II T cell proliferation (Extended Data Fig. 9j). These results suggest that uPAR^+^ epithelial cells that accumulate in the small intestine during natural aging can express MHC-II and potentially uptake and present antigens to T cells, conceivably contributing to inflammaging.

Taken together, our data suggest that targeting only immune cells with uPAR CAR T cells in aged mice is not sufficient to ameliorate intestinal aging phenotypes. Conversely, epithelial uPAR^+^ cells have decreased cell intrinsic stemness, and their direct targeting with uPAR CAR T cells is sufficient to rejuvenate the activity of aged ISCs, suggesting that uPAR^+^ epithelial cells are key drivers of intestinal aging and associated inflammation and dysfunction.

## Discussion

Despite its highly regenerative capacity and importance as a regulator of whole-body physiology, little is known about the cellular basis of intestinal aging. Herein, we identify the accumulation of intestinal uPAR^+^ cells during physiological aging in mice and humans, which are characterized by the absence of proliferative markers and enrichment in markers of DNA repair and inflammation. Their elimination through in vivo treatment with uPAR CAR T cells led to improvements in intestinal epithelial barrier integrity and tissue fitness in aging. We found that ablating uPAR^+^ cells with uPAR CAR T cells increased the number of ISCs, proliferation and regenerative capacity of the aged intestinal epithelium. Moreover, uPAR^+^ cell ablation reduced age-associated intestinal inflammation and dysbiosis. Finally, transplantation and in vitro experiments suggest that it is the ablation of uPAR^+^ epithelial cells, rather than immune cells, that primarily drive these improvements. Together, these findings demonstrate that the accumulation of uPAR^+^ epithelial cells in the small intestines of mice and humans are key drivers of aging-associated inflammation and intestinal dysfunction. This can be reversed by the administration of uPAR CAR T cells in aged mice or prevented through their continuous elimination during aging.

Beyond defects in regeneration, the aged intestinal niche is characterized by chronic inflammation and defects in mucosal immunity. In our work we found that elimination of uPAR^+^ cells resulted in decreased markers of inflammaging and improved overall mucosal immune function. Interestingly, we found that intestinal epithelial uPAR^+^ cells are able to present antigen to CD4 T cells through MHC-II, although the identity of the antigens that are being presented and the direct in vivo functional consequences remain to be elucidated. IFNy produced by CD4 T cells can trigger the upregulation of MHC-II expression in aged ISCs, promoting inflammaging and loss of intestinal homeostasis^46,47^. Whether IFNy is also responsible for the upregulation of MHC-II on intestinal uPAR^+^ cells remains to be studied. Interestingly, elimination of uPAR^+^ cells resulted in a decrease in the levels of IFNy in the aged intestinal epithelium. Future work on this area could provide further insights into the interplay among tissue injury, inflammation and the induction of uPAR expression.

Finally, this study also provides proof-of-principle for immune cell engineering as a regenerative medicine modality. Unlike other regenerative therapeutics that rely on dietary interventions or continuous dosing of small molecules^5–7,9–13^; uPAR CAR T cells can mediate long-term beneficial effects after a single low-dose administration, presenting a prolife that may enhance patients’ adherence and quality of life. Whether these benefits extend to other stem cell niches remains to be determined. Future CAR T approaches could also explore alternative cell surface targets that are upregulated in specific dysfunctional niches or utilize different immune cell types or delivery routes to enhance therapeutic precision^48^. Further work is needed to assess the safety profile and a potential side effect of promoting tissue regeneration could be an increased tumorigenesis, but it is noteworthy that none of the mice treated with uPAR CAR T in this study, including those treated for over 18 months, developed intestinal malignancies. The use of combinatorial strategies^49^ and/or the incorporation of safety switches^50^ could provide versatile strategies to address these possible toxicities. Altogether, the high efficacy of uPAR CAR T cells to improve intestinal fitness in aging mice underscores the potential of immune-based cellular therapy to promote tissue regeneration.

## Methods

### Mice and drug treatments

All mouse experiments were approved by CSHL Internal Animal Care and Use Committee (protocol number 21-4). All relevant animal use guidelines and ethical regulations were followed. Mice were maintained under specific pathogen-free conditions. Housing was on a 12-h/12-h light/dark cycle under standard temperature and humidity of approximately 18–24 °C and 40–60%, respectively. The following mice from The Jackson Laboratory were used: 3-month-old C57BL/6 J mice (000664), 18- to 20-month-old C57BL/6 J mice (000664) and 6-week-old and 18-month-old B6.SJL-Ptrc^a^ Pepc^b^/BoyJ (CD45.1 mice) (002014) and 17- to 25-month-old Lgr-EGFP-IRES-creERT2 mice (008875). Mice of both sexes were used at 3 months of age and 18–20 months of age for the aging experiments and females of 6–10 weeks old for T cell isolation. For dasatinib and quercetin treatments, mice were administered dasatinib (50 mg kg^−1^) (Sigma, CDS023389-25MG) and quercetin (Sigma, Q4951-100G) resuspended in 10% ethanol, 30% polyethylene glycol and 60% Phosal by oral gavage twice a week for 6 weeks as described previously^21^. For abdominal irradiation experiments, mice were irradiated locally once with 15 Gy in the abdomen with the help of a lead protector device covering the rest of the body. For Edu administration, Edu (Thermo Fisher Scientific, A10044) was injected at 0.5 mg kg^−1^ 4 h before euthanasia as described elsewhere^44^. For BODIPY 500/510, C_1_,C_12_ administration, BODIPY 500/510, C_1_,C_12_ (Thermo Fisher Scientific, D3823) was administered by oral gavage (10 μl g^−1^ body weight) 2 h before euthanasia as described previously^51^. For adoptive T cell transfer, mice were treated with one intraperitoneal injection of cyclophosphamide 200 mg kg^−1^ (Sigma, C0768) 18 h before T cell injection as described elsewhere^25^. For bone marrow transplant, mice were treated with three consecutive daily doses of busulfan 30 mg kg^−1^ (Sigma, B2635-10G) a week before. Ovalbumin-Texas red (Thermo Fisher Scientific, O23021) was administered by oral gavage at 1 mg kg^−1^ 1 h before euthanasia as described previously^52^. Immunization with ovalbumin was performed by administering 1 mg OVA (Sigma, A7641) by oral gavage three times at 1-week intervals as described elswehere^53^. Mice were kept in group housing. Mice had free access to food and water and were fed PicoLab Rodent Diet 20 (LabDiet). Mice were randomly assigned to the experimental groups.

### Human samples

De-identified human normal colon tissue samples were obtained from colon adenocarcinoma patients (female 91 years of age, female 51 years of age and male 83 years of age) undergoing surgical resection procedures at Huntington Hospital, with written informed consent. All human studies complied with all relevant guidelines and ethical regulations and were reviewed and approved by the Northwell Health Biospecimen Repository (protocol number 1810).

### Intestinal crypt isolation and flow cytometry

As previously reported^6,44^, whole small intestine was removed, washed with cold PBS ^− /−^, opened laterally and cut into 3–5 mm fragments. Pieces were washed multiple times with ice cold PBS − /− until clean, washed 2–3 with ice cold 1X PBS, and incubated in PBS/EDTA (7.5 mM) with mild agitation for 30 min at 4 C. Crypts were then mechanically separated from the connective tissue by shaking, and filtered through a 70-μm mesh into a 50 ml conical tube to remove villus material and tissue fragments. Crypts were removed from this step for crypt culture experiments and embedded in Matrigel (Corning 356231 growth factor reduced) with crypt culture media. Crypts were removed from this step for protein isolation. For EpCAM^+^ cell isolation, the crypt suspensions were dissociated to individual cells with TrypLE Express (Thermo Fisher Scientific, 12604039) and stained for flow cytometry. Epithelial cells were isolated as SYTOX^−^, CD45^−^ EpCAM^+^ with a BD FACS Aria II SORP cell sorter into supplemented crypt culture medium for culture. For experiments with Lgr5-GFP mice, cells were sorted as GFP^−^, CD24^+^ or GFP^+^ uPAR^+^ or GFP^+^ uPAR^−^ with a BD FACS Aria II SORP cell sorter into supplemented crypt culture medium for culture. uPAR^+^ and uPAR^−^ populations were isolated as DAPI^−^, uPAR^+/−^ with a SONY cell sorter(SH800S). For immune phenotyping, dissociated crypt suspensions were stained for flow cytometry. For this, Fc receptors were blocked using FcR blocking reagent, mouse (Miltenyi Biotec). The following fluorophore-conjugated antibodies were used: PE-uPAR (FAB531P, R&D systems, lot ABLH0521021), AF700-uPAR (FAB531N, R&D systems, lot AFNL0122081), BV785-CD45.1 (110743, BioLegend, lot B319039), AF488-CD3 (100210, BioLegend, lot B364217), BUV395-CD4 (563790, BD Biosciences, lot 1165066), PE-Cy7-CD8 (100722, BioLegend, lot B282418), BV421-CD62L (104435, BioLegend, lot B283191), APCCy7-CD44 (560568, BD Biosciences, lot 1083068), BV650-LAG3 (125227, BioLegend, lot B333220), BV510-PD1 (BioLegend, 135241, lot B342120), BV605-CD25 (102035, BioLegend, lot B354812), APC-EpCAM (118214, BioLegend, lot B280290), FITC-CD45 (103102, BioLegend, lot 2041142), FITC-MHC-II (11-5321-82, Invitrogen, lot 2442242), PE-CD153 (12-1531-82, Invitrogen, lot 2504402), BV510-PD1 (135241, BioLegend, lot B342120), BV711-CD45.2 (109847, BioLegend, lot B348415), PE-Texas red-CD28 (102124, BioLegend, lot B376397), BUV737-KLRG1 (741812, BD Biosciences, lot 2327039), BUV395-CD11b (563553, BD Horizon, lot 3346840), PerCP-Cy5.5-CD11c (117328, BioLegend, lot B332774), APC-Cy7-Ly6C (128026, BioLegend, B309226), BV605-Ly6G (563005, BD Biosciences, lot 3187156), PE-TR-F4/80 (61-4801-82, Invitrogen, 2452260), AF700-uPAR (FAB531N, R&D systems, lot 1656339), PE-CD19 (553786, BD Pharminogen, 1312594), BV650-CD19 (563235, BD Biosciences,4213621), PE-Cy7-CD3 (100220, BioLegend, B401339), BV711-CD24 (101851, BioLegend, B446985). Ghost UV 450 Viability Dye (13-0868-T100, Tonbo Biosciences lot D0868083018133) or SYTOX Blue dead cell stain (Thermo Fisher Scientific, S34857; lot2491422) or DAPI (Sigma, 32670-5MG-F) was used as viability dye. Flow cytometry was performed on a LSRFortessa instrument (BD Biosciences), and data were analyzed using FlowJo (TreeStar).

### Bone marrow isolation and flow cytometry

For whole bone marrow isolation, single-cell suspensions were prepared by crushing the femurs, tibias, and iliac crests of each mouse using a mortar and pestle on ice. The resulting suspensions were filtered through a 70 μm cell strainer, and red blood cells were lysed using ACK lysing buffer (Gibco) for 5 min on ice. Lysis was quenched with a twofold volume of FACS buffer (1× PBS supplemented with 2% FBS), followed by centrifugation at 300 × *g* for 5 min at 4 °C. To block Fc receptors, cells were incubated with FcR blocking reagent, mouse (Miltenyi Biotec) for 10 min at 4 °C. CD45.1^−^ and CD45.2^+^ populations were isolated as DAPI^−^, CD45.1^−^ and CD45.2^+^ with a SONY cell sorter (SH800S). For immune phenotyping, single-cell suspensions were stained for flow cytometry. For this, Fc receptors were blocked using FcR blocking reagent, mouse (Miltenyi Biotec). The following fluorophore-conjugated antibodies were used: BV785-CD45.1 (110743, BioLegend, lot B319039), BV711-CD45.2 (109847, BioLegend, lot B348415), BV650-CD19 (563235, BD Biosciences, 4213621), PE-Cy7-CD3 (100220, BioLegend, B401339), PerCP-Cy5.5-CD11c (117328, BioLegend, lot B332774), BUV395-CD11b (563553, BD Horizon, lot 3346840), APC-Cy7-Ly6C (128026, BioLegend, B309226), BV605-Ly6G (563005, BD Biosciences, lot 3187156), FITC-MHC-II (11-5321-82, Invitrogen, lot 2442242). Ghost UV 450 Viability Dye (13-0868-T100, Tonbo Biosciences lot D0868083018133) or DAPI (Sigma, 32670-5MG-F) was used as viability dye. Flow cytometry was performed on a LSRFortessa instrument (BD Biosciences), and data were analyzed using FlowJo (TreeStar).

### Peripheral blood isolation and flow cytometry

Peripheral blood was collected via submandibular puncture using an 18 G needle. A 15 μl aliquot of whole blood was lysed in ACK lysing buffer (Gibco) for 5 min on ice. Lysis was quenched with a twofold volume of FACS buffer, followed by centrifugation at 300 × g for 5 min at 4 °C.

Fc receptors were subsequently blocked using FcR blocking reagent, mouse (Miltenyi Biotec). The following fluorophore-conjugated antibodies were used: BV785-CD45.1 (110743, BioLegend, lot B319039), BV711-CD45.2 (109847, BioLegend, lot B348415), BV650-CD19 (563235, BD Biosciences, 4213621), PE-Cy7-CD3 (100220, BioLegend, B401339), PerCP-Cy5.5-CD11c (117328, BioLegend, lot B332774), BUV395-CD11b (563553, BD Horizon, lot 3346840), APC-Cy7-Ly6C (128026, BioLegend, B309226), BV605-Ly6G (563005, BD Biosciences, lot 3187156), FITC-MHC-II (11-5321-82, Invitrogen, lot 2442242). Ghost UV 450 Viability Dye (13-0868-T100, Tonbo Biosciences, lot D0868083018133) or DAPI (Sigma, 32670-5MG-F) was used as viability dye. Flow cytometry was performed on a LSRFortessa instrument (BD Biosciences), and data were analyzed using FlowJo (TreeStar).

### scRNA-seq

Three scRNA-seq experiments were conducted in mice: 1) whole small intestine from young and old UT or uPAR CAR T (m.uPAR-m.28z)-treated mice 6 weeks after treatment, 2) sorted uPAR^+^ or uPAR^−^ cells from aged intestines, and 3) whole small intestine from young (3 months) mice were treated with UT or uPAR CAR T cells 15 months after treatment. In the CAR T treatment dataset a total of four replicates per treatment groups (uPAR & UT) with stratified sampling of age and sex (two males and two females per age and treatment group). For the uPAR^+^ or uPAR^−^ dataset there were two replicates per sample totaling two females and two males. For the dataset from young mice treated with uPAR CAR T cells for 15 months there is one replicate each from the UT- and uPAR-treated groups. Additionally, we analyzed publicly available human scRNA-seq data from a previous study^35^. Analysis of the human data was conducted on the duodenum, jejunum and ileum of young (aged 25–30 years) and old (aged 65–70 years) patients and intestinal immune cells from young (aged 25–30 years) and old (aged 65–70 years) patients. Single-cell datasets for each experiment were independently assessed for data quality following the guidelines described previously^54,55^. After QC, Seurat (v4.0.3 (ref. ^56^)) was used for normalization, graph-based clustering and differential expression analysis. Each dataset was normalized using *SCTransform* and the 2,500 most variable genes were identified with *SelectIntegrationFeatures*. The CAR T cell treatment dataset was integrated by sample into a singular dataset via using the *PrepSCTIntegration, FindIntegrationAnchors, and IntegrateData* functions^57^. Likewise, the uPAR sorted dataset was integrated by the sex of the samples using the *PrepSCTIntegration, FindIntegrationAnchors*, and *IntegrateData* functions to retain differences in clustering between treatment conditions. MAGIC imputation was conducted on integrated data to impute missing values and account for technical noise^58^. *RunPCA* was implemented on the integrated datasets to identify the top principal components that were used for UMAP analysis and clustering. Louvain clustering at a resolution of 1 was implemented. Clusters were labeled in accordance with expression levels of intestinal epithelial subtype signatures identified previously^33^. Scores were assigned calculating the average z-score of the average expression of the genes in each cell. Wilcoxon rank-sum tests to determine if differences in metagene scores was significant was conducted using the *wilcox.test* function in stats (v4.1.0, R Core Team, 2021). Senescent cells were identified by first creating metagene scores for senescence using the signatures described previously^34^. Cells expressing the metagene signature greater than the inflection point of the distribution of expression were deemed to be senescent. Differential expression analysis was conducted using the *FindMarkers* function with the MAST method to correct for covariates such as sex and evaluate differences within the transcriptome^59^. Gene set enrichment analysis was conducted on differentially expressed genes (of either logFC >0.1 or < −0.1 and adjusted *P* <.05) using the enrichR package (v3.2 (ref. ^60^)). Monocle3 (v1.3.4 ref. ^61^)) was used for pseudotime trajectory analysis of the CAR T treatment dataset.

### Organoid culture for crypts and isolated cells

Isolated crypts were counted and embedded in Matrigel (Corning, 356231 growth factor reduced) at 5–10 crypts μl^−1^ and cultured in a modified form of medium as described previously^62^. Unless otherwise stated, Advanced DMEM (Thermo Fisher Scientific, 12491023) with 10% penicillin/streptomycin (GeminiBio, 400-109) was supplemented by EGF 40 ng ml^−1^ (Peprotech, 315-09), Noggin 50 ng ml^−1^ (Peprotech, 250-38), R-spondin 62.5 ng ml^−1^ (Peprotech, 315-32), *N*-acetyl-L-cysteine 1 μM (Sigma-Aldrich, A9165), N2 1X (Gibco, 17502-048), B27 1X (Gibco, 17504-044), CHIR-99021 10 μM (Tocris, 4423) and Y-27632 dihydrochloride monohydrate 20 ng ml^−1^ (Tocris, 1254). Then, 25 μl drops of Matrigel with crypts were plated onto a flat-bottom 48-well plate (Corning, 3524) and allowed to solidify for 5–6 min in a 37 °C incubator. Five hundred microliters of crypt culture medium were then overlaid onto the Matrigel, changed every other day and maintained at 37 °C in fully humidified chambers containing 5% CO_2_. Clonogenicity (colony-forming efficiency) was calculated by plating 50–300 crypts per well and assessing organoid formation 3–7 days or as specified after initiation of cultures. Organoids were propagated as previously described^6,44^. For secondary subculture experiments, primary organoids were separated for a duration of 6 min using TrypLE Express (Thermo Fisher Scientific, 12604039) at a temperature of 37 °C. The resulting dissociated single cells were counted and plated equally in Matrigel and left to solidify. GFP⁺ Lgr5⁺ ISCs (ISCs; CD45⁻, EpCAM⁺, CD24^−^, Lgr5-GFP⁺) were sorted by flow cytometry into uPAR⁺ and uPAR⁻ populations in equal numbers, along with Paneth cells (CD45⁻, EpCAM⁺, CD24⁺). Cells were centrifuged at 300 × *g* for 5 min. A total of 50,000 uPAR⁺ or uPAR⁻ ISCs were resuspended in crypt culture medium containing an equal number of Paneth cells and seeded into 25–30 μl Matrigel (Corning) per well, in a flat-bottom 24-well plate. After solidification, Matrigel was supplemented with crypt medium containing 1 μM Jagged (Anaspec). Crypt culture medium was replaced every 2–3 days, and organoids were quantified on day 8 of culture. The culture medium was refreshed every other day with fresh crypt media, and the organoids were maintained at 37 °C in a fully humidified chamber with 5% CO_2_. Several random, non-overlapping brightfield images were acquired from each well using a Nikon Eclipse TS2 microscope equipped with 4×/0.13 NA and 10×/0.25 NA objective lenses. Organoids were imaged directly in their culture wells. Quantification was performed using Fiji software as described previously^4,6,63–71^, and viability was assessed based on morphological criteria, including lumen appearance and overall structural integrity. Organoids exhibiting a darkened lumen and disrupted or collapsed structure were classified as nonviable. In contrast, organoids with an intact structure and clear, well-defined borders were considered viable. The classification was guided by both image-based metrics and visual inspection to ensure accurate distinction between live and dead organoids as performed previously^4,6,63–71^.

### Organoid transduction

Organoids derived from 18-month-old mice were mechanically dislodged from the culture plate using cold Cell Recovery Solution (Corning) and transferred to a 1.5 ml microcentrifuge tube. After removing the CRS and Matrigel, organoid pellets were incubated with 500 μl TrypLE Express at 37 °C. To facilitate dissociation, the organoids were pipetted gently every 2 min during the incubation. The reaction was then quenched with crypt culture medium. For viral transduction, 10 μg ml^−1^ Polybrene (Sigma-Aldrich) was added to crypt culture medium containing the blasticidin-resistant Cas9-expressing lentivirus (Addgene, catalog no. 52962) and mixed gently. Dissociated intestinal organoids were resuspended in the virus-containing medium and transferred to a 48-well plate. Plates were centrifuged at 600 × *g* for 1 h at room temperature and subsequently incubated at 37 °C for 4 h. After incubation, cells were embedded in Matrigel and plated for culture. To select for organoids transduced with the blasticidin resistance cassette, crypt culture medium containing 1 μg ml^−1^ blasticidin was added 3 days after transduction. After selection and confirmation of Cas9 expression in aged intestinal organoids, Cas9⁺ organoids were transduced with a puromycin-resistant lentiviral plasmid (pUSEPR (U6-sgRNA-EFS-Puro-P2A-TurboRFP)^72^ encoding either a non-targeting control sgRNA or sgRNAs targeting *Plaur* (guide 1: AAGGATGAGGACTACACCCG or guide 2: AACTACACCCACTGCAATGG), as described above, to generate control and *Plaur* KO organoids. To select for successfully transduced cells, crypt culture medium containing 1 μg ml^−1^ puromycin was added 3 days after transduction.

### Organoid bulk RNA sequencing

Young (3 months) and old (18 months) mice were treated with 0.5 × 10^6^ untransduced T cells (UT) or uPAR CAR T cells (m.uPAR-m.28z). Mice were harvested 8 weeks after infusion, and organoids were generated from their intestinal crypts. 5 days after generation, organoids were harvested as described above and subjected to bulk RNA sequencing. The resulting RNA-seq data were analyzed by removing adaptor sequences using CutAdapt^73^. RNA-seq reads were then aligned with STAR^74^, and the transcript count was quantified using featureCounts^75^ to generate a raw-count matrix. Differential gene expression analysis and adjustment for multiple comparisons were performed using the DESeq2 package^76^ between experimental conditions, with at least two independent biological replicates per condition, implemented in R (http://cran.r-project.org/). Genes were determined to be differentially expressed based on a greater than twofold change in gene expression with an adjusted *P* value of less than 0.05. For lollipop visualization of enriched pathways, differentially expressed genes were calculated with DESeq2 using the method ashr^77^ for LFC shrinkage and preranked based on log2FC. Preranked genes were analyzed using GSEA^78^ to calculate enriched pathways based on Molecular Signature Database

Hallmark 2025 signatures and an ISC signature^33^. Graphs were generated by the GSEA program or plotted in R using the ggplot2 package. *P* values in GSEA were calculated using a two-sided non-parametric permutation test, where phenotype labels were randomly permuted 1,000 times to generate a null distribution of enrichment scores, with false discovery rate correction applied for multiple comparisons. The normalized enrichment score accounts for differences in gene set size by normalizing the enrichment score to the mean enrichment score of the same gene set across all permutations and is calculated using a weighted Kolmogorov-Smirnov test.

### Histological analysis

Tissues and organoids were fixed overnight in 10% formalin, embedded in paraffin and cut into 5-μm sections. Sections were subjected to hematoxylin and eosin (H&E) staining. Immunohistochemical staining was performed following standard protocols. The following primary antibodies were used: uPAR (AF534, R&D systems, lot DCL0724051, cleaved caspase-3 (9664S, Cell Signaling Technology, lot 22), EpCAM (93790S, Cell Signaling Technology, lot 3), Olfm4 (39141S, Cell Signaling Technology, lot 4), F4/80 (70076S, Cell Signaling Technology, lot 9), p21 (ab107099, Abcam,1067675-2), E-cadherin (AF748,R&D, CYG0424111). The following secondary antibodies were used: HRP Horse anti-goat IgG (MP-7405, Vector Laboratories, lot ZJ0718), HRP horse anti-rabbit IgG (MP-7401, Vector Laboratories, lot ZH0609), AF488-donkey anti-rabbit IgG (A21206, Invitrogen, 2376850) and AF594-donkey anti-goat (A11058, Invitrogen, 2445414), AF488-donkey anti-rat IgG (A21208, Invitrogen, 2482958) and AF488-donkey anti-goat IgG (A11055, Invitrogen, 2747580). For detection of EdU, the Click-iT Plus EdU Alexa Fluor 647 and 488 Imaging Kit (Thermo Fisher, C10640 or C10637) was used.

### Multiplex immunofluorescence

Multiplex immunofluorescence was performed on 5-μm FFPE human tissue sections. Sections were deparaffinized, rehydrated, and subjected to two-step antigen retrieval using Citrate buffer (pH 6.0), followed by Tris-EDTA buffer (pH 9.0). Slides were then blocked in PBS containing 3% bovine serum albumin (BSA), stained with DAPI, and imaged to capture baseline autofluorescence. Staining was performed manually in sequential cycles using a ClickWell slide holder with a sealed chamber. Each cycle consisted of primary antibody incubation, followed by secondary antibody staining when necessary. All washes were performed using TBST, and all rounds of imaging and slide storage were done in a solution of PBS with 50% glycerol. Slides were scanned after each round using the CellDive instrument (Leica), which provided automated imaging, autofluorescence subtraction, image registration to baseline DAPI, and field-of-view stitching using the CellDive image acquisition and processing software. After imaging, fluorophore inactivation was performed using 0.1 M Na_2_CO_3_ with 3% H_2_O_2_ for 15 min at room temperature. This staining-imaging-inactivation cycle was repeated for a total of 8 markers, using DAPI, FITC, Cy3, Cy5 and Cy7 channels for acquisition. Staining quality and fluorescence removal were verified after each round. The fully stitched images were imported into HALO image analysis software (Indica Labs) for analysis. Cell segmentation was performed using the ‘traditional’ nuclear segmentation option, with analysis settings optimized for each staining category. The following antibodies were used: uPAR (AF807, R&D), AF555-Ki-67 (558617, BD Biosciences), AF647-γH2A.X (ab195189, Abcam), AF488-E-cadherin (3199S, Cell Signaling Technology), AF647-p21 (8587S, Cell Signaling Technology), AF488-CD31 (42777, Cell Signaling Technology), AF555-CD45 (19744, Cell Signaling Technology), AF750 cleaved caspase-3 (97774S, Cell Signaling Technology), and AF555-donkey anti goal (A21432, Invitrogen).

### SA-β-gal staining

SA-β-gal staining was performed as previously described^79^ at pH 5.5 for mouse tissues. Specifically, fresh frozen tissue sections were fixed with 0.5% glutaraldehyde in phosphate-buffered saline (PBS) for 15 min, washed with PBS supplemented with 1 mM MgCl_2_ and stained for 5–8 h in PBS containing 1 mM MgCl_2_, 1 mg ml^−1^ X-gal, 5 mM potassium ferricyanide and 5 mM potassium ferrocyanide. Tissue sections were counterstained with eosin. Three fields per section were counted with ImageJ and averaged to quantify the percentage of SA-β-gal^+^ area per field. For SPiDER-β-gal experiments, intestinal crypts were dissociated into single-cell suspensions and cultured with SPiDER-β-gal substrate at 37 C for 30 min at 37 °C according to manufacturer’s instructions (Dojindo, SG02-10). Subsequently, cells were stained with PE-uPAR (FAB531P, R&D systems, lot ABLH0521021). DAPI (Sigma, 32670-5MG-F) was used as viability dye. Flow cytometry was performed on a LSRFortessa instrument (BD Biosciences), and data were analyzed using FlowJo (TreeStar).

### Intestinal permeability assay

Mice were fasted for 6 h before starting the test and a pre-test plasma sample was collected after this time. Subsequently, mice were administered by oral gavage 150 μl of 80 mg ml^−1^ FITC-Dextran (4 kDa) (Sigma-Aldrich; FD4-250 mg). Plasma sample collection was repeated 4 h post-gavage. The pre- and post-plasma samples were diluted 1:10 in PBS and a total volume of 100 μl transferred to a black 96-well plate. Pre- and post-plasma fluorescence levels were determined in a plate reader at 530 nm with excitation at 485 nm. Results were normalized to the average of the control group.

### Isolation, expansion and transduction of mouse T cells

B6.SJL-Ptrc^a^ Pepc^b^/BoyJ(CD45.1 mice) were euthanized and spleens were collected. After tissue dissection and red blood cell lysis, primary mouse T cells were purified using the mouse Pan T cell Isolation Kit (Miltenyi Biotec, 130-095-130). Purified T cells were cultured in RPMI-1640 (Invitrogen, 11-875-085) supplemented with 10% FBS (Corning, 35-010-CV), 10 mM HEPES (Thermo Fisher Scientific, 15630080), 2 mM L-glutamine (Thermo Fisher Scientific, 25030164), MEM non-essential amino acids 1x (Thermo Fisher Scientific, 11140076), 55 µM β-mercaptoethanol (Thermo Fisher Scientific, 21985023), 1 mM sodium pyruvate (Thermo Fisher Scientific, 11360070), 100 IU ml^−1^ recombinant human IL-2 (Proleukin, Novartis) and mouse anti-CD3/28 Dynabeads (Gibco, 11452D) at a bead/cell ratio of 1:2. T cells were spinoculated with retroviral supernatant collected from Phoenix-ECO cells 24 h after initial T cell activation as described previously^80,81^ and used for functional analysis 3–4 days later.

### Genetic modification of T cells

The mouse SFG γ-retroviral m.uPAR-m28z plasmid has been described previously^25^ and was obtained from Memorial Sloan Kettering Cancer Center. In this construct the anti-mouse uPAR single-chain variable fragment is preceded by a mouse CD8A leader peptide and followed by the Myc-tag sequence (EQKLISEEDL), mouse CD28 transmembrane and intracellular domain and mouse CD3z intracellular domain^80,81^. A plasmid encoding the SFGγ retroviral vector was used to transfect gpg29 fibroblasts (H29) to generate VSV-G pseudotyped retroviral supernatants, which were used to construct stable retrovirus-producing cell lines as described elsewhere^80,82^.

### Antigen presentation experiments

Were performed as described previously^45^. In brief, 5 × 10^3^ sort-purified CD45^−^ EpCAM^+^uPAR^+^ or uPAR^−^ cells were cultured with 5 × 10^4^ OT-II T cells in the organoid culture medium described above (without Matrigel), with or without 15 μg ml^−1^ ovalbumin peptide (Anaspec, AS-27024) at 37 °C for 72 h. T cell proliferation was assessed using the CellTrace Violet proliferation kit (Thermo Fisher Scientific, C34557) per manufacturer’s instructions.

### Cytokine analysis

Intestinal crypts were isolated and protein was extracted in lysis buffer (20 mM Tris HCl (pH 7.5), 0.5% Tween 20 (Sigma, P1379) and 150 mM NaCl, 1:100 protease inhibitor (Thermo Fisher Scientific, 87786). Protein concentration was determined with BCA protein assay kit (Thermo Fisher Scientific, 23228). Cytokine measurement on the protein lysates was performed by Eve Technologies.

### Detection of granzyme B or IgA levels or lipocalin-2 levels

Levels of granzyme B, total IgA, anti-OVA IgA and lipocalin-2 from mouse plasma were evaluated by ELISA according to the manufacturer’s protocols (Abcam, ab238265, granzyme B; Thermo Fisher Scientific, BMS6029, granzyme B; Abcam, ab157717, total; Chondrex, 3018, anti-OVA; and R&D Systems, MLCN20, lipocalin-2).

### Taxonomic microbiota analysis/metagenomics

Metagenomics sequencing analysis of fecal samples was performed by Transnetyx as described previously^83^. Briefly, fresh mouse fecal samples were placed in barcoded sample collection tubes containing DNA stabilization buffer and shipped to Transnetyx where DNA extraction, library preparation, sequencing, data preprocessing and preliminary analysis were performed. These analyses involved first aligning individual sequences using 31 base k-mers to the One Codex Database. Sequencing artifacts are then filtered out of the sample based on the relative frequency of unique k-mers per sample. Finally, the relative abundance of microbial species was estimated as a function of the number of reads covering that genome and the genome’s size. Bray-Curtis dissimilarity was utilized for beta diversity estimation. PCoA analysis and PERMANOVA testing were conducted on Bray-Curtis dissimilarity matrices of microbe abundance across samples using the ggvegan package in R.

### Quantification, reproducibility and statistical analysis

Unless specified, statistical analysis was performed using GraphPad Prism v.6.0 or 7.0 (GraphPad software). Data distribution was assumed to be normal but this was not formally tested. Flow cytometry data was analyzed with FlowJo 10.8.1 (FlowJo). Images were analyzed with Image J-Fiji (NIH). No statistical methods were used to predetermine sample size in the mouse studies, and no method of randomization was used to assign mice to treatment groups, but groups were balanced by sex. No data were excluded from the analysis except for flow cytometry experiments if the viability of the sample was less than 30%. Experiments were repeated in replicates and/or from different subjects in independent experiments. Information on experimental repetition and replicates is provided in the figure legends. All attempts at replication were successful. Mouse conditions were observed by an operator who was blinded to the treatment groups in addition to the main investigator who was not blind to group allocation. Data collection and analysis were not performed blind to the conditions of the experiments. Figures were prepared with BioRender.com for scientific illustrations and Illustrator CC 2022 (Adobe).

### Reporting summary

Further information on research design is available in the Nature Portfolio Reporting Summary linked to this article.

## Supplementary information

Reporting Summary

## Source data

Source data. This is a single excel file containing all source data with clearly named tabs for each figure and extended data figure item.

## Figures and Tables

**Extended Data Fig. 10:**
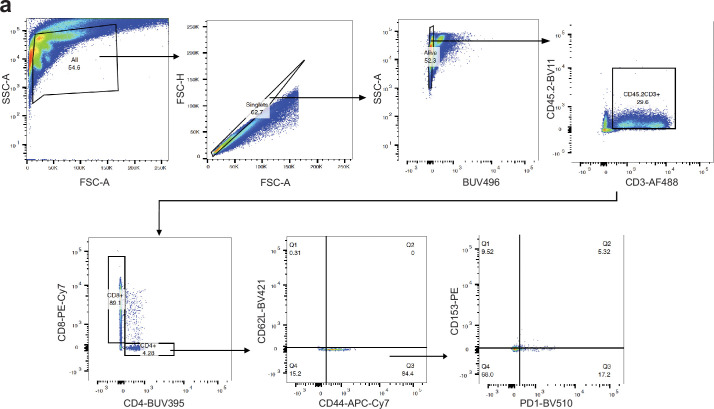
Gating strategy. **a**, Representative flow cytometry staining of senescent endogenous T cells (CD153+PD1+) from CD4+CD44+CD62L- T cells in the intestinal crypts of a young untransduced (UT) treated mice weeks after cell infusion (n=4 mice per group). Shown are results of 1 independent experiment.

## Data Availability

scRNA-seq and bulk RNA-seq data presented in this study have been deposited in the Gene Expression Ominus database under accession number GSE233431. Metagenomics data were deposited in the Sequence Read Archive under accession number PRJNA1117419. Source data are provided with this paper. Requests for materials and any additional data should be addressed to the corresponding authors.

## Code availability

Original code can be found in the following GitHub repositories: https://github.com/Vyoming/Regen_CAR-T.git and https://github.com/AmorLab/Nature-Aging-Intestinal-Senescence.
