# Supplementary material for: Anti-uPAR CAR T cells reverse and prevent aging-associated defects in intestinal regeneration and fitness
Source: Nat Aging. 2025 Nov 25;6(1):108–26. doi: 10.1038/s43587-025-01022-w (PMC12823409; doi:10.1038/s43587-025-01022-w)
Supplement: Supplementary file 1 — Reporting Summary [file 43587_2025_1022_MOESM1_ESM.pdf]

nature portfolio

Corresponding author(s): Corina Amor Vegas

Last updated by author(s): Oct 22, 2025

Reporting Summary

Nature Portfolio wishes to improve the reproducibility of the work that we publish. This form provides structure for consistency and transparency in reporting. For further information on Nature Portfolio policies, see our Editorial Policies and the Editorial Policy Checklist.

Statistics

For all statistical analyses, confirm that the following items are present in the figure legend, table legend, main text, or Methods section.

n/a	Confirmed	
☐	☒	The exact sample size (*n*) for each experimental group/condition, given as a discrete number and unit of measurement
☐	☒	A statement on whether measurements were taken from distinct samples or whether the same sample was measured repeatedly
☐	☒	The statistical test(s) used AND whether they are one- or two-sided<br>*Only common tests should be described solely by name; describe more complex techniques in the Methods section.*
☒	☐	A description of all covariates tested
☒	☐	A description of any assumptions or corrections, such as tests of normality and adjustment for multiple comparisons
☐	☒	A full description of the statistical parameters including central tendency (e.g. means) or other basic estimates (e.g. regression coefficient) AND variation (e.g. standard deviation) or associated estimates of uncertainty (e.g. confidence intervals)
☐	☒	For null hypothesis testing, the test statistic (e.g. *F*, *t*, *r*) with confidence intervals, effect sizes, degrees of freedom and *P* value noted<br>*Give P values as exact values whenever suitable.*
☒	☐	For Bayesian analysis, information on the choice of priors and Markov chain Monte Carlo settings
☒	☐	For hierarchical and complex designs, identification of the appropriate level for tests and full reporting of outcomes
☒	☐	Estimates of effect sizes (e.g. Cohen's *d*, Pearson's *r*), indicating how they were calculated

Our web collection on statistics for biologists contains articles on many of the points above.

Software and code

Policy information about availability of computer code

Data collection	BD-Fortessa cytometer, ZEISS Axio Observer microscopy, CellDive instrument, Illumina NextSeq 500.
Data analysis	FlowJo 10.8.1, GraphPad Prism V.9.3.1, Image J-Fiji, R V4.3.2,CellDive image acquisition and processing software, Microsoft Excel for Mac V16.77, Seurat V4.0.3., R Core Team 2021 v4.1.0, enrichRpackage v3.2, Monocle3 v1.3.4.<br>Original code can be found in the following GitHub repositories: https://github.com/Vyoming/Regen_CAR-T.git and https://github.com/AmorLab/Nature-Aging-Intestinal-Senescence.

For manuscripts utilizing custom algorithms or software that are central to the research but not yet described in published literature, software must be made available to editors and reviewers. We strongly encourage code deposition in a community repository (e.g. GitHub). See the Nature Portfolio guidelines for submitting code & software for further information.

Data

Policy information about availability of data

All manuscripts must include a data availability statement. This statement should provide the following information, where applicable:

- Accession codes, unique identifiers, or web links for publicly available datasets
- A description of any restrictions on data availability
- For clinical datasets or third party data, please ensure that the statement adheres to our policy

scRNA-seq and bulk RNAseq data presented in this study is deposited in the Gene Expression Ominus database under accession number GSE233431. Metagenomics

data was deposited in the Sequence Read Archive under accession number PRJNA1117419. Source data is provided with this paper. Requests for materials and any additional data should be addressed to the corresponding authors.

Human research participants

Policy information about studies involving human research participants and Sex and Gender in Research.

Reporting on sex and gender	We employed de-identified human normal colon tissue samples that were obtained from colon adenocarcinoma patients (female 91 years of age, female 51 years of age and male 83 years of age) with written informed consent undergoing surgical resection procedures at Huntington Hospital
Population characteristics	De-identified human samples from (female 91 years of age, female 51 years of age and male 83 years of age) with a diagnosis of colon adenocarcinoma were obtained.
Recruitment	We obtained the samples through the Northwell Health Biospecimen Repository.
Ethics oversight	All human studies complied with all relevant guidelines and ethical regulations, and were reviewed and approved by the Northwell Health Biospecimen Repository (Protocol number: 1810).

Note that full information on the approval of the study protocol must also be provided in the manuscript.

Field-specific reporting

Please select the one below that is the best fit for your research. If you are not sure, read the appropriate sections before making your selection.

☒ Life sciences ☐ Behavioural & social sciences ☐ Ecological, evolutionary & environmental sciences

For a reference copy of the document with all sections, see nature.com/documents/nr-reporting-summary-flat.pdf

Life sciences study design

All studies must disclose on these points even when the disclosure is negative.

Sample size	No statistical methods were used to pre-determine sample size. Sample sizes were estimated based on preliminary experiments, with an effort to achieve a minimum of n=3 mice per treatment group which proved to be sufficient to reproducibly observe a statistical significant difference.
Data exclusions	For flow cytometry experiments samples with less than 30% of viability were excluded from the analysis.
Replication	Experiments were repeated in replicates and/or from different subjects in independent experiments. Information on experimental repetition and replicates is provided in the figure legends. All attempts at replication were successful.
Randomization	No method of randomization was used to assign mice to treatment groups, but groups were balanced by sex
Blinding	Mouse conditions were observed by an operator who was blinded to the treatment groups in addition to the main investigator who was not blind to group allocation. Data analysis was not performed in a blinded fashion. Data analysis are based on objectively measurable data (eg: scRNA sequencing, cell counts in flow cytometry).

Reporting for specific materials, systems and methods

We require information from authors about some types of materials, experimental systems and methods used in many studies. Here, indicate whether each material, system or method listed is relevant to your study. If you are not sure if a list item applies to your research, read the appropriate section before selecting a response.

Materials & experimental systems

n/a	Involved in the study	
☐	☒	Antibodies
☒	☐	Eukaryotic cell lines
☒	☐	Palaeontology and archaeology
☐	☒	Animals and other organisms
☒	☐	Clinical data
☒	☐	Dual use research of concern

Methods

n/a	Involved in the study	
☒	☐	ChIP-seq
☐	☒	Flow cytometry
☒	☐	MRI-based neuroimaging

Antibodies

Antibodies used

The following fluorophore-conjugated antibodies were used for flow cytometry: PE-uPAR (FAB531P, R&D systems, lot ABLH0521021,1:50), AF700-uPAR (FAB531N, R&D systems, lot AFNL0122081, 1:50), BV785-CD45.1 (110743, BioLegend, lot B319039, 1:100), AF488-CD3 (100210, BioLegend, lot B364217, 1:100), BUV395-CD4 (563790, BD Biosciences, lot 1165066, 1:50), PECy7-CD8 (100722, BioLegend, lot B282418, 1:50), BV421-CD62L (104435, BioLegend, lot B283191, 1:50), APCCy7-CD44 (560568,BD Biosciences, lot 1083068, 1:100), BV650-LAG3 (125227, BioLegend, lot B333220, 1:100), BV510-PD1 (BioLegend, 135241, lot B342120, 1:50), BV605-CD25 (102035, BioLegend, lot B354812, 1:100), APC-Epcam (118214, Biolegend,lot B280290, 1:100), FITC-CD45 (103102, BioLegend, lot 2041142, 1:100), FITC-MHCII (11-5321-82, Invitrogen, lot 2442242, 1:100), PE-CD153 (12-1531-82, Invitrogen, lot 2504402, 1:200), BV510-PD1 (135241, BioLegend, lot B342120, 1:50), BV711-CD45.2 (109847, BioLegend, lot B348415, 1:100), PE-Texas red-CD28 (102124, BioLegend, lot B376397, 1:100), BUV737-KLRG1 (741812, BD Biosciences, lot 2327039, 1:100), BUV395-CD11b (563553, BD Horizon, lot 3346840, 1:50), PerCP-Cy5.5-CD11c (117328, Biolegend, lot B332774 1:100), APC-Cy7-Ly6C (128026, Biolegend, B309226, 1:100), BV605-Ly6G (563005, BD Biosciences, lot 3187156, 1:100), PE-TR-F4/80 (61-4801-82, Invitrogen, 2452260, 1:100), AF700-uPAR (FAB531N, R&D systems, lot 1656339, 1:50), PE-CD19 (553786, BD Pharminogen, 1312594, 1:100), BV650-CD19 (563235, BD Biosciences,4213621, 1:100), PE-Cy7-CD3 (100220, Biolegend,B401339, 1:50), BV711-CD24 (101851, Biolegend, B446985, 1:100). Ghost UV 450 Viability Dye (13-0868-T100, Tonbo Biosciences lot D0868083018133, 1ul/ml) or SYTOX Blue dead cell stain (Thermo Fisher Scientific, S34857; lot2491422, 1ul/ml) or DAPI (Sigma, 32670-5MG-F, 1:1000) was used as viability dye. For multiplex immunofluorescence the following antibodies were used: uPAR (AF807, R&D, 1:500), AF555-Ki-67 (558617, BD Bioscience, 1:50), AF647-gH2A.X (ab195189, Abcam, 1:100), AF488-E-cadherin (3199S, Cell Signaling Technology, 1:200), AF647-p21 (8587S, Cell Signaling Technology, 1:150), AF488-CD31 (42777, Cell Signaling Technology, 1:100), AF555-CD45 (19744, Cell Signaling Technology, 1:100), AF750-Cleaved caspase 3 (97774S, Cell Signaling Technology, 1:100), AF555-donkey anti goal (A21432, Invitrogen, 1:1000). For histology, the following primary antibodies were used: uPAR (AF534, R&D systems, lot DCL0724051, 1:50), Cleaved caspase 3 (9664S, Cell Signaling Technology, lot 22,1:2000), Epcam (93790S, Cell Signaling Technology, lot 3, 1:150), Olfm4 (39141S, Cell Signaling Technology, lot 4, 1:200), F4/80 (70076S, Cell Signaling Technology, lot 9, 1:125), p21 (ab107099, Abcam,1067675-2, 1:100), E-cadherin (AF748,R&D, CYG0424111, 10ug/ml). The following secondary antibodies were used: HRP Horse anti-goat IgG (MP-7405, Vector Laboratories, lot ZJ0718), HRP Horse anti-rabbit IgG (MP-7401, Vector Laboratories, lot ZH0609), AF488-donkey Anti rabbit IgG (A21206, Invitrogen, 2376850, 1:500) and AF594-donkey anti goat (A11058, Invitrogen, 2445414, 1:500), AF488-donkey Anti rat IgG (A21208, Invitrogen, 2482958, 1:500), AF488-donkey anti goat IgG (A11055, Invitrogen, 2747580, 1:500).

Validation

All used antibodies were titrated. All the antibodies are validated for use in flow cytometry or immunohistochemistry or immunofluorescence. Data are available at the manufacturer's website. All used antibodies are commercially available. The following fluorophore-conjugated antibodies were used for flow cytometry: PE-uPAR (FAB531P, R&D systems, lot ABLH0521021,1:50), AF700-uPAR (FAB531N, R&D systems, lot AFNL0122081, 1:50), BV785-CD45.1 (110743, BioLegend, lot B319039, 1:100), AF488-CD3 (100210, BioLegend, lot B364217, 1:100), BUV395-CD4 (563790, BD Biosciences, lot 1165066, 1:50), PECy7-CD8 (100722, BioLegend, lot B282418, 1:50), BV421-CD62L (104435, BioLegend, lot B283191, 1:50), APCCy7-CD44 (560568,BD Biosciences, lot 1083068, 1:100), BV650-LAG3 (125227, BioLegend, lot B333220, 1:100), BV510-PD1 (BioLegend, 135241, lot B342120, 1:50), BV605-CD25 (102035, BioLegend, lot B354812, 1:100), APC-Epcam (118214, Biolegend,lot B280290, 1:100), FITC-CD45 (103102, BioLegend, lot 2041142, 1:100), FITC-MHCII (11-5321-82, Invitrogen, lot 2442242, 1:100), PE-CD153 (12-1531-82, Invitrogen, lot 2504402, 1:200), BV510-PD1 (135241, BioLegend, lot B342120, 1:50), BV711-CD45.2 (109847, BioLegend, lot B348415, 1:100), PE-Texas red-CD28 (102124, BioLegend, lot B376397, 1:100), BUV737-KLRG1 (741812, BD Biosciences, lot 2327039, 1:100), BUV395-CD11b (563553, BD Horizon, lot 3346840, 1:50), PerCP-Cy5.5-CD11c (117328, Biolegend, lot B332774 1:100), APC-Cy7-Ly6C (128026, Biolegend, B309226, 1:100), BV605-Ly6G (563005, BD Biosciences, lot 3187156, 1:100), PE-TR-F4/80 (61-4801-82, Invitrogen, 2452260, 1:100), AF700-uPAR (FAB531N, R&D systems, lot 1656339, 1:50), PE-CD19 (553786, BD Pharminogen, 1312594, 1:100), BV650-CD19 (563235, BD Biosciences,4213621, 1:100), PE-Cy7-CD3 (100220, Biolegend,B401339, 1:50), BV711-CD24 (101851, Biolegend, B446985, 1:100). Ghost UV 450 Viability Dye (13-0868-T100, Tonbo Biosciences lot D0868083018133, 1ul/ml) or SYTOX Blue dead cell stain (Thermo Fisher Scientific, S34857; lot2491422, 1ul/ml) or DAPI (Sigma, 32670-5MG-F, 1:1000) was used as viability dye. For multiplex immunofluorescence the following antibodies were used: uPAR (AF807, R&D, 1:500), AF555-Ki-67 (558617, BD Bioscience, 1:50), AF647-gH2A.X (ab195189, Abcam, 1:100), AF488-E-cadherin (3199S, Cell Signaling Technology, 1:200), AF647-p21 (8587S, Cell Signaling Technology, 1:150), AF488-CD31 (42777, Cell Signaling Technology, 1:100), AF555-CD45 (19744, Cell Signaling Technology, 1:100), AF750-Cleaved caspase 3 (97774S, Cell Signaling Technology, 1:100), AF555-donkey anti goal (A21432, Invitrogen, 1:1000). For histology, the following primary antibodies were used: uPAR (AF534, R&D systems, lot DCL0724051, 1:50), Cleaved caspase 3 (9664S, Cell Signaling Technology, lot 22,1:2000), Epcam (93790S, Cell Signaling Technology, lot 3, 1:150), Olfm4 (39141S, Cell Signaling Technology, lot 4, 1:200), F4/80 (70076S, Cell Signaling Technology, lot 9, 1:125), p21 (ab107099, Abcam,1067675-2, 1:100), E-cadherin (AF748,R&D, CYG0424111, 10ug/ml). The following secondary antibodies were used: HRP Horse anti-goat IgG (MP-7405, Vector Laboratories, lot ZJ0718), HRP Horse anti-rabbit IgG (MP-7401, Vector Laboratories, lot ZH0609), AF488-donkey Anti rabbit IgG (A21206, Invitrogen, 2376850, 1:500) and AF594-donkey anti goat (A11058, Invitrogen, 2445414, 1:500), AF488-donkey Anti rat IgG (A21208, Invitrogen, 2482958, 1:500), AF488-donkey anti goat IgG (A11055, Invitrogen, 2747580, 1:500).

Animals and other research organisms

Policy information about studies involving animals; ARRIVE guidelines recommended for reporting animal research, and Sex and Gender in Research

Laboratory animals

The following mice from The Jackson Laboratory were used: 3-month-old C57BL/6J mice (000664), 18 to 20-month-old C57BL/6J mice (000664) and 6-week-old and 18 months old B6.SJL-Ptrca Pepcb/BoyJ (CD45.1 mice) (002014), 17-25 month old Lgr-EGFP-IRES-creERT2 mice (008875). Housing was on a 12-h–12-h light–dark cycle under standard temperature and humidity of approximately 18–24 °C and 40–60%, respectively.

Wild animals

This study did not involve wild animals.

Reporting on sex

Mice of both sexes were used at 3 months of age and 18-20 months of age for the aging experiments and females of 6-10 weeks old for T cell isolation.

Field-collected samples

This study did not involve samples collected from the field.

Ethics oversight

Cold Spring Harbor Laboratory (CSHL) Internal Animal Care and Use Committee.

Note that full information on the approval of the study protocol must also be provided in the manuscript.

Flow Cytometry

Plots

Confirm that:

☒ The axis labels state the marker and fluorochrome used (e.g. CD4-FITC).

☒ The axis scales are clearly visible. Include numbers along axes only for bottom left plot of group (a 'group' is an analysis of identical markers).

☒ All plots are contour plots with outliers or pseudocolor plots.

☒ A numerical value for number of cells or percentage (with statistics) is provided.

Methodology

Sample preparation

Whole small intestine was removed, washed with cold PBS–/–, opened laterally and cut into 3-5mm fragments. Pieces were washed multiple times with ice cold PBS–/– until clean, washed 2-3 with ice cold 1X PBS, and incubated in PBS/EDTA (7.5mM) with mild agitation for 30 minutes at 4C. Crypts were then mechanically separated from the connective tissue by shaking, and filtered through a 70-µm mesh into a 50 mL conical tube to remove villus material and tissue fragments. Dissociated crypt suspensions were stained for flow cytometry. For this, Fc receptors were blocked using FcR blocking reagent, mouse (Miltenyi Biotec). The following fluorophore-conjugated antibodies were used: PE-uPAR (FAB531P, R&D systems, lot ABLH0521021), AF700-uPAR (FAB531N, R&D systems, lot AFNL0122081), BV785-CD45.1 (110743, BioLegend, lot B319039), AF488-CD3 (100210, BioLegend, lot B364217), BUV395-CD4 (563790, BD Biosciences, lot 1165066), PECy7-CD8 (100722, BioLegend, lot B282418), BV421-CD62L (104435, BioLegend, lot B283191), APCCy7-CD44 (560568,BD Biosciences, lot 1083068), BV650-LAG3 (125227, BioLegend, lot B333220), BV510-PD1 (BioLegend, 135241, lot B342120), BV605-CD25 (102035, BioLegend, lot B354812), APC-Epcam (118214, Biolegend,lot B280290), FITC-CD45 (103102, BioLegend, lot 2041142), FITC-MHCII (11-5321-82, Invitrogen, lot 2442242), PE-CD153 (12-1531-82, Invitrogen, lot 2504402), BV510-PD1 (135241, BioLegend, lot B342120), BV711-CD45.2 (109847, BioLegend, lot B348415), PE-Texas red-CD28 (102124, BioLegend, lot B376397), BUV737-KLRG1 (741812, BD Biosciences, lot 2327039), BUV395-CD11b (563553, BD Horizon, lot 3346840), PerCP-Cy5.5-CD11c (117328, Biolegend, lot B332774), APC-Cy7-Ly6C (128026, Biolegend, B309226), BV605-Ly6G (563005, BD Biosciences, lot 3187156), PE-TR-F4/80 (61-4801-82, Invitrogen, 2452260), AF700-uPAR (FAB531N, R&D systems, lot 1656339), PE-CD19 (553786, BD Pharminogen, 1312594), BV650-CD19 (563235, BD Biosciences,4213621), PE-Cy7-CD3 (100220, Biolegend,B401339), BV711-CD24 (101851, Biolegend, B446985). Ghost UV 450 Viability Dye (13-0868-T100, Tonbo Biosciences lot D0868083018133) or SYTOX Blue dead cell stain (Thermo Fisher Scientific, S34857; lot2491422) or DAPI (Sigma, 32670-5MG-F) was used as viability dye. Flow cytometry was performed on a LSRFortessa instrument (BD Biosciences), and data were analyzed using FlowJo (TreeStar).

For whole bone marrow isolation, single-cell suspensions were prepared by crushing the femurs, tibias, and iliac crests of each mouse using a mortar and pestle on ice. The resulting suspensions were filtered through a 70µm cell strainer, and red blood cells were lysed using ACK lysing buffer (Gibco) for 5 minutes on ice. Lysis was quenched with a twofold volume of FACS buffer (1× PBS supplemented with 2% FBS), followed by centrifugation at 300 × g for 5 minutes at 4°C. To block Fc receptors, cells were incubated with FcR blocking reagent, mouse (Miltenyi Biotec) for 10 minutes at 4°C. For immune phenotyping, single cell suspensions were stained for flow cytometry. For this, Fc receptors were blocked using FcR blocking reagent, mouse (Miltenyi Biotec). The following fluorophore-conjugated antibodies were used: BV785-CD45.1 (110743, BioLegend, lot B319039), BV711-CD45.2 (109847, BioLegend, lot B348415), BV650-CD19 (563235, BD Biosciences,4213621), PE-Cy7-CD3 (100220, Biolegend,B401339), PerCP-Cy5.5-CD11c (117328, Biolegend, lot B332774), BUV395-CD11b (563553, BD Horizon, lot 3346840), APC-Cy7-Ly6C (128026, Biolegend, B309226), BV605-Ly6G (563005, BD Biosciences, lot 3187156), FITC-MHCII (11-5321-82, Invitrogen, lot 2442242). Ghost UV 450 Viability Dye (13-0868-T100, Tonbo Biosciences lot D0868083018133) or DAPI (Sigma, 32670-5MG-F) was used as viability dye. Flow cytometry was performed on a LSRFortessa instrument (BD Biosciences), and data were analyzed using FlowJo (TreeStar).

Peripheral blood was collected via submandibular puncture using an 18G needle. A 15µL aliquot of whole blood was lysed in ACK lysing buffer (Gibco) for 5 minutes on ice. Lysis was quenched with a twofold volume of FACS buffer, followed by centrifugation at 300 × g for 5 minutes at 4°C.
Fc receptors were subsequently blocked using FcR blocking reagent, mouse (Miltenyi Biotec). The following fluorophore-conjugated antibodies were used: BV785-CD45.1 (110743, BioLegend, lot B319039), BV711-CD45.2 (109847, BioLegend, lot B348415), BV650-CD19 (563235, BD Biosciences,4213621), PE-Cy7-CD3 (100220, Biolegend,B401339), PerCP-Cy5.5-CD11c (117328, Biolegend, lot B332774), BUV395-CD11b (563553, BD Horizon, lot 3346840), APC-Cy7-Ly6C (128026, Biolegend, B309226), BV605-Ly6G (563005, BD Biosciences, lot 3187156), FITC-MHCII (11-5321-82, Invitrogen, lot 2442242). Ghost UV 450 Viability Dye (13-0868-T100, Tonbo Biosciences lot D0868083018133) or DAPI (Sigma, 32670-5MG-F) was used as viability dye. Flow cytometry was performed on a LSRFortessa instrument (BD Biosciences), and data were analyzed using FlowJo (TreeStar).

Instrument	LSRFortessa instrument (BD Biosciences), SONY cell sorter(SH800S).
Software	Collection: FACS DIVA.<br>Analysis: Flowjo 10.8.1
Cell population abundance	The purity was verified by flow cytometry.
Gating strategy	The starting cell population was gated on a SSC-A/FSC-A plot. Cell siglets were identified by FSC/SSC gating. Positive/Negative populations were determined by FMO controls.

☒ Tick this box to confirm that a figure exemplifying the gating strategy is provided in the Supplementary Information.
